# Translational assessment of nursing care models for oncology patients with cardiovascular risk factors

**DOI:** 10.3389/fcvm.2026.1834086

**Published:** 2026-10-19

**Authors:** Huili Du, Jing Wang, Yuguang Xing

**Affiliations:** 1 Surgical Oncology, Xinxiang Central Hospital, Xinxiang, Henan, China; 2 Pharmaceutical Department, Xinxiang Central Hospital, Xinxiang, Henan, China

**Keywords:** cardiovascular risk factors, counterfactual event planner, individualized treatment effect, nursing care strategies, oncology patients

## Abstract

**Introduction:**

The objective of this study was to estimate patient-specific effects of alternative acute nursing care strategies for critically ill oncology patients with cardiovascular risk factors using routinely collected electronic health record data. Existing nursing care evaluations primarily characterize population-level associations or predict observed outcomes, but they do not estimate the potential outcomes of individual patients under standard and enhanced acute care while accounting for evolving clinical states and predictive uncertainty.

**Methods:**

We developed the Counterfactual Event Planner to estimate individualized treatment effects and compare alternative nursing care strategies through clinically constrained trajectory modeling and probabilistic outcome estimation. The proposed framework integrates the Manifold Based Decision Model, Agent based Contextual Navigator, and Probabilistic Outcome Synthesizer to construct clinically meaningful latent representations, explore context-aware intervention trajectories, and estimate potential outcome distributions. Furthermore, Counterfactual Pacing Strategy and Bayesian Outcome Updating enable dynamic trajectory simulation and uncertainty-aware outcome refinement, improving the reliability of individualized treatment effect estimation.

**Results and discussion:**

Extensive experiments on the MIMIC-IV and eICU Collaborative Research Database demonstrate that the proposed framework consistently outperforms representative statistical, neural, and causal inference baselines in individualized treatment effect estimation, policy evaluation, and outcome prediction while maintaining robust cross-hospital generalization. The proposed framework provides an effective and generalizable solution for counterfactual evaluation of nursing care strategies, supporting personalized clinical decision making for oncology patients with cardiovascular risk factors.

## 1 Introduction

Oncology patients with cardiovascular risk factors require nursing care that integrates cancer-treatment support with cardiovascular monitoring and risk management. These concurrent clinical needs may affect treatment tolerance, symptom burden, care coordination, and long-term outcomes. This dual burden underscores the need for translational assessment of nursing care models that can integrate cardiovascular risk management into oncology care (1). Moreover, the rising cost and complexity of cancer care require innovative models that improve patient outcomes while supporting efficient and coordinated care delivery (2). Therefore, exploring and refining nursing care models for this patient population is timely and essential for advancing high quality, patient centered oncology care (3, 4). The initial focus on oncology patients with pre-existing cardiovascular risk factors was selected for both clinical and methodological reasons. Clinically, this population is particularly vulnerable because baseline cardiovascular conditions may interact with anticancer treatment, acute physiological stress, and supportive care requirements, thereby increasing the need for timely nursing assessment and coordinated risk management. Methodologically, selecting a population with a higher expected burden of cardiovascular events provides a clearer setting for estimating heterogeneous care effects, comparing alternative nursing strategies, and evaluating counterfactual predictions while reducing the influence of severe outcome sparsity. This targeted population should therefore be interpreted as an initial validation group rather than as the only population that may benefit from cardiovascular monitoring. Cardiovascular toxicity may also occur among patients who are initially classified as having low cardiovascular risk, and baseline assessment remains relevant across the broader population receiving anticancer therapy. Future studies should extend the framework to all patients undergoing anticancer treatment, include low, moderate, high, and very high cardiovascular risk groups, and examine whether monitoring intensity and nursing interventions can be adapted according to baseline risk and changes observed during treatment.

Traditional oncology care models have emphasized shared decision making, psychosocial support, and coordinated service delivery. Shared decision making is particularly important in cancer care because patients and clinicians must jointly consider treatment benefits, risks, preferences, and supportive care needs (5). Comprehensive cancer care also requires attention to psychosocial needs, communication, continuity, and multidisciplinary collaboration (6). However, conventional care models may have limited ability to dynamically integrate heterogeneous clinical data, changing cardiovascular risk profiles, and evolving nursing observations during acute care. The increasing availability of electronic health records, care coordination tools, and implementation science frameworks has created new opportunities for data-informed oncology nursing assessment. Care coordination measurement and implementation studies have emphasized the importance of structured clinical information, multidisciplinary communication, and context-sensitive care delivery for cancer patients with complex comorbidities (7, 8). These developments support the need for computational frameworks that can use real-world clinical records to evaluate individualized nursing care strategies while remaining interpretable and clinically meaningful. Technology-enabled oncology care has further expanded the possibilities for remote monitoring, supportive care delivery, and individualized assessment. Digital health, telehealth, and mobile health studies have shown that technology can support symptom monitoring, patient communication, and supportive cancer care across different clinical settings (9–11). Nevertheless, technology-drive care models still face challenges related to data quality, interpretability, workflow integration, patient equity, and clinical acceptability (12–14). These challenges motivate the development of a framework that combines clinical knowledge, structured patient data, counterfactual reasoning, and uncertainty-aware outcome estimation.

Based on the aforementioned limitations, this study proposes a Counterfactual Event Planner for the translational assessment of nursing care models in oncology patients with cardiovascular risk factors. The proposed framework integrates manifold based patient representation, context-aware intervention navigation, probabilistic outcome synthesis, counterfactual trajectory simulation, and Bayesian outcome updating. By estimating potential outcomes under alternative nursing care strategies, the framework aims to support individualized assessment, risk-adjusted care planning, and clinical decision support for oncology patients with cardiovascular risk factors.

The contributions are summarized as follows:

- The proposed framework introduces counterfactual outcome estimation into the translational assessment of oncology nursing care models for patients with cardiovascular risk factors.
- The method integrates clinically grounded patient representations, contextual intervention navigation, probabilistic outcome synthesis, and uncertainty-aware updating to support individualized care strategy evaluation.
- Experiments on real critical care electronic health record cohorts demonstrate improved performance in treatment effect estimation, policy evaluation, outcome prediction, and cross-hospital external validation.

## 2 Related work

### 2.1 Integrative care models in oncology

Integrative care models in oncology represent a comprehensive approach to patient management, particularly for patients with complex comorbidities and supportive care needs. high-quality cancer care requires coordinated, patient centered, and multidisciplinary service delivery that addresses diagnosis, treatment planning, communication, symptom management, survivorship, and follow up care (3, 4). Care coordination is a central component of this process because oncology patients often move across multiple care settings and clinical specialties (7). The integration of cardiology and oncology care is clinically important because cancer treatment may interact with cardiovascular risk factors and increase the need for proactive monitoring and coordinated management. Survivorship care recommendations and palliative care integration studies further emphasize the value of multidisciplinary planning, continuity of care, symptom management, and individualized support for patients living with advanced or complex cancer conditions (15–17). Patient and caregiver experiences also indicate that co management models can improve communication and care coordination when multiple specialties are involved (18). The implementation of integrative care models requires practical strategies that facilitate communication and collaboration among different professionals. Implementation science provides useful guidance for identifying barriers, aligning care pathways with local context, and integrating new care coordination interventions into routine oncology practice (1, 8). In diverse healthcare settings, integrative care must also consider structural barriers, inequities, and differences in access to cancer services (12, 13, 19–21). These studies suggest that nursing care models should be evaluated not only by predictive performance, but also by clinical relevance, workflow compatibility, equity, and patient centered value.

### 2.2 Cardio oncology nursing interventions

Nursing interventions are essential for oncology patients with cardiovascular risk factors because nurses frequently provide continuous assessment, symptom monitoring, patient education, care coordination, and communication across multidisciplinary teams. In supportive and palliative oncology care, nursing related interventions often address symptom burden, treatment tolerance, nutrition, anxiety, psychosocial needs, and continuity of care (15, 22, 23). These responsibilities are especially relevant for patients with comorbid cardiovascular conditions because changes in physiological status may require timely monitoring and escalation. Preventive and supportive interventions may include patient education, early recognition of deterioration, monitoring of vital signs, review of treatment-related symptoms, coordination with physicians, and reinforcement of individualized care plans. Digital and telehealth-based supportive cancer care can further extend nursing assessment by enabling remote communication, follow up, and symptom monitoring (9, 10). Mobile health and web-based interventions have also been studied in cancer care for monitoring, psychosocial support, and quality of life improvement (11, 24). Clinical relevance in nursing care model development requires the participation of care stakeholders. Patient centered oncology initiatives and advisory council experiences highlight the value of incorporating patient, caregiver, and clinician perspectives when designing care pathways or decision support tools (25). New care models for specific oncology populations also suggest that multidisciplinary input is important for aligning computational or organizational innovations with real clinical needs (26). Therefore, the evaluation of nursing care models should include both quantitative performance and stakeholder acceptability.

### 2.3 Technology-drive care innovations

Technology-drive care innovations are transforming oncology care by supporting remote monitoring, digital communication, symptom assessment, and care coordination. Telehealth-based supportive cancer care has been widely discussed as a strategy for improving access, reducing unnecessary visits, and supporting ongoing communication between patients and healthcare teams (9). Broader reviews of digital health and telehealth in cancer care further show that digital tools can support symptom tracking, patient engagement, remote consultation, and supportive care delivery (10). Mobile health applications and web-based interventions provide additional opportunities for individualized oncology care. Mobile health studies in breast cancer care have examined digital tools for monitoring, education, communication, and supportive management (11, 27). Web-based dyadic interventions have also been evaluated for psychological distress, depression, stress, and quality of life among persons with cancer and informal caregivers (24). These studies indicate that technology can extend oncology care beyond traditional encounters and provide additional data streams for patient assessment. At the same time, technology-drive care requires careful consideration of privacy, data quality, interpretability, access, and equity. Cancer care equity studies show that structural barriers, geography, social determinants, and population specific needs can influence access to care and the effectiveness of digital or coordinated interventions (12–14, 19–21). Computational nursing care models should be developed with attention to transparency, clinical oversight, subgroup fairness, and implementation feasibility. These considerations are particularly important when automated decision support is used for high risk oncology patients with cardiovascular comorbidities.

### 2.4 Counterfactual learning and causal decision support in clinical care

The increasing availability of electronic health records has created opportunities to move beyond conventional outcome prediction toward individualized estimation of the effects of alternative clinical strategies. Counterfactual learning is particularly relevant in this setting because observational clinical records contain outcomes only for the care actually received, whereas the outcomes that would have occurred under alternative strategies remain unobserved. Representation learning has therefore been introduced into counterfactual inference to reduce systematic differences between treatment groups and improve the estimation of individual treatment effects from observational data (28, 29). Balanced representations can reduce discrepancies between treated and control populations while preserving outcome-related information, providing an important foundation for heterogeneous treatment effect estimation and precision-oriented clinical decision making (29). Deep latent-variable models further extend this direction by representing latent confounding structures through observed proxy variables, allowing causal effects to be estimated in settings where relevant clinical factors may not be directly measured (30). Statistical and neural causal inference approaches have subsequently expanded the capacity to estimate heterogeneous treatment responses from high-dimensional clinical observations. Flexible forest-based methods enable nonparametric estimation of heterogeneous effects while supporting uncertainty quantification and subgroup-specific analysis (31). Neural treatment effect models combine shared patient representations with treatment-specific outcome estimation, while propensity-related objectives and targeted regularization can further improve causal adjustment in observational data (32). These developments demonstrate the potential of machine learning for individualized treatment effect estimation, but many existing approaches primarily model static baseline covariates and a single treatment decision, which may be insufficient for acute care environments characterized by continuously changing physiological states, nursing observations, and intervention requirements. Longitudinal counterfactual modeling addresses this limitation by considering treatment effects over sequential clinical trajectories. Sequence-to-sequence causal models can construct treatment-invariant representations of patient histories and estimate potential outcomes under alternative treatment sequences while accounting for time-varying confounding (33). Such approaches are particularly relevant to critical care because physiological measurements, treatment decisions, monitoring activities, and supportive interventions evolve continuously and may jointly influence subsequent patient states. However, reliable clinical decision support requires not only accurate counterfactual estimation but also transparent validation, uncertainty assessment, human oversight, and careful integration into clinical workflows. Early-stage evaluation guidance for artificial intelligence-based clinical decision support emphasizes clinical utility, safety, human factors, and preparation for prospective evaluation before large-scale implementation (34). Transparent reporting and external validation are also essential for determining whether machine-learning-based clinical prediction systems are reproducible, interpretable, and applicable across different patient populations and healthcare settings (35). Despite these advances, a methodological gap remains between general treatment effect estimation and the translational assessment of nursing care for oncology patients with cardiovascular risk factors. Existing causal learning frameworks generally emphasize treatment effect estimation itself, whereas nursing care evaluation requires the simultaneous representation of heterogeneous oncological and cardiovascular conditions, temporally evolving nursing observations, clinically feasible care trajectories, and uncertainty associated with predicted outcomes. A unified framework that integrates patient representation, contextual intervention navigation, probabilistic outcome estimation, dynamic counterfactual trajectory simulation, and uncertainty-aware updating may therefore provide a more suitable foundation for individualized nursing care assessment in complex oncology populations.

## 3 Method

### 3.1 Overview

This section delineates our methodological framework for the translational assessment of nursing care models tailored for oncology patients with cardiovascular risk factors. The research aims to establish a comprehensive framework that integrates and evaluates nursing care models, ensuring clinical relevance and adaptability to the specific needs of oncology patients at heightened risk of cardiovascular complications. Central to our approach is the Counterfactual Event Planner, which serves as the pivotal model for our analysis.

The framework uses structured electronic health record variables collected during the first 24 h of intensive care admission to estimate patient-specific outcomes under standard and enhanced acute nursing care. The Manifold-Based Decision Model encodes demographic, oncological, cardiovascular, physiological, treatment, and nursing variables into a clinically informed latent patient state. The Agent-Based Contextual Navigator then generates feasible intervention trajectories under the predefined care strategies, while the Probabilistic Outcome Synthesizer estimates the corresponding potential outcome distributions. Counterfactual Pacing Strategy imposes temporal and clinical constraints on the simulated trajectories, and Bayesian Outcome Updating refines outcome estimates and predictive uncertainty as additional observations are incorporated. The differences between the estimated potential outcomes are used to quantify individualized nursing care effects and compare alternative care strategies.

### 3.2 Preliminaries

This section formalizes the problem of evaluating nursing care models for oncology patients with cardiovascular risk factors. The objective is to create a framework that assesses and optimizes nursing care interventions by considering the interaction between oncology and cardiovascular conditions. This requires integrating diverse data sources and applying advanced modeling techniques to predict patient outcomes under various care scenarios.

Let P represent the set of oncology patients, each described by a feature vector $x_{i}\in R^{d}$, where i∈P. These features encompass demographic data, clinical history, and specific cardiovascular risk factors. The aim is to evaluate the impact of different nursing care models, denoted by $M=\{M_{1},M_{2},\ldots ,M_{k}\}$, on patient outcomes.

In the implemented clinical task, $x_{i}$ is not an abstract input vector. It is constructed from structured electronic health record variables recorded during the first twenty four hours of intensive care admission. It contains demographic characteristics, cancer diagnosis information, previous disease history, cardiovascular risk factors, vital signs, laboratory measurements, early treatment records, nursing observations, monitoring frequency, supportive care records, and missingness indicators. Cardiovascular risk factors include documented hypertension, diabetes, coronary artery disease, heart failure, arrhythmia, abnormal blood pressure, abnormal heart rate, oxygen saturation abnormality, and cardiovascular diagnosis records. Oncology related variables include cancer diagnosis codes, cancer-related admission information, treatment history when available, and clinical severity indicators associated with tumor care. Thus, the formulation is calibrated to observed oncology and cardiovascular variables rather than being used as a generic mathematical template.

The outcome of interest for a patient i under a care model $M_{j}$ is $Y_{ij}$, a random variable representing the clinical outcome, such as survival rate or quality of life. The challenge lies in estimating the counterfactual outcomes $Y_{ij}$ for each patient under different care models, necessitating the modeling of the potential outcomes framework.

The potential outcome for patient i under model $M_{j}$ is denoted as $Y_{i}(M_{j})$. The observed outcome is $Y_{i}=Y_{i}(M_{\;j^{*}})$, where $M_{\;j^{*}}$ is the care model actually received by the patient. The task is to estimate the unobserved potential outcomes $Y_{i}(M_{j})$ for all $j\neq j^{*}$.

In this study, $M_{j}$ corresponds to predefined acute nursing care strategies. The binary implementation includes standard acute care and enhanced acute care. Enhanced acute care is assigned when the composite score of early nursing intensity, physiological monitoring frequency, and supportive care reaches the median threshold calculated from the MIMIC IV training cohort. The clinical outcome vector includes intensive care unit mortality, in hospital mortality, cardiovascular adverse events, intensive care unit length of stay, and clinical recovery status. Mortality outcomes are derived from recorded death indicators and discharge status. Cardiovascular adverse events are extracted from diagnosis records and documented clinical events. Intensive care unit length of stay is calculated from admission and discharge timestamps, and clinical recovery status is determined from physiological improvement, organ support withdrawal, and discharge disposition.

To address this, a latent variable $Z_{i}$ is introduced to capture the underlying health state of patient i. The relationship between the observed features, the latent health state, and the potential outcomes is modeled as: ?>

$$Z_{i}=f(x_{i},\epsilon_{i})$$ (1)

$$Y_{i}(M_{j})=g(Z_{i},M_{j},\eta_{ij})$$ (2)

where f and g are deterministic functions, and $\epsilon_{i}$ and $\eta_{ij}$ are stochastic components representing unobserved heterogeneity and model-specific effects, respectively.

The Counterfactual Event Planner framework estimates these potential outcomes by utilizing the Manifold Based Decision Model, which maps the high-dimensional feature space into a lower dimensional manifold that captures the essential variability in patient health states. This is formalized as:

$$z_{i}=\phi (x_{i})$$ (3)

where $\phi \;:\;R^{d}\to R^{m}$ is a nonlinear mapping function.

The Agent based Contextual Navigator uses these encoded representations to simulate patient trajectories under different care models. The probabilistic nature of the outcomes is captured by the Probabilistic Outcome Synthesizer, which models the distribution of potential outcomes as:

$$P(Y_{i}(M_{j})|z_{i})=N(\mu_{ij},\sigma_{ij}^{2})$$ (4)

where $\mu_{ij}$ and $\sigma_{ij}^{2}$ are the mean and variance of the outcome distribution, respectively, conditioned on the encoded patient state.

The clinical correspondence of the mathematical quantities is summarized in Table 1. This mapping clarifies that Equations (1) to (4) are estimated from clinical oncology and cardiovascular data, and that each latent or probabilistic term has a direct implementation in the nursing intervention assessment task.

**Table 1:** Clinical correspondence of variables and equations.

Equation or variable	Model quantity	Clinical meaning and implementation
$x_{i}$	Patient feature vector	Demographics, cancer diagnosis information, cardiovascular risk factors, vital signs, laboratory measurements, treatment records, nursing observations, monitoring frequency, supportive care, and missingness indicators from the first twenty four hours of intensive care admission.
$M_{j}$	Nursing care model	Standard acute care or enhanced acute care defined by nursing intensity, physiological monitoring frequency, and supportive care measures.
$Z_{i}$ and $z_{i}$	Latent health state	Patient health representation that summarizes oncological condition, cardiovascular burden, physiological instability, and nursing care context.
$Y_{i}(M_{j})$	Potential outcome	Predicted clinical outcome under a candidate nursing care strategy, including mortality, cardiovascular adverse events, length of stay, and recovery status.
$\epsilon_{i}$	Unobserved heterogeneity	Latent factors such as undocumented frailty, unmeasured disease severity, measurement noise, and incomplete clinical documentation.
$\eta_{ij}$	strategy-specific response variation	Variation in patient response under a particular care strategy, including heterogeneity in treatment tolerance and nursing intervention benefit.
$\mu_{ij},\sigma_{ij}^{2}$	Outcome distribution parameters	Estimated mean and uncertainty of patient outcomes under each care strategy. Binary endpoints use Bernoulli likelihoods, and continuous endpoints use Gaussian likelihoods with learned means and variances.

The integration of these components facilitates the estimation of counterfactual outcomes and the evaluation of nursing care models’ effectiveness. The following sections will explore the specifics of the Counterfactual Event Planner, detailing the construction of the Manifold Based Decision Model, the design of the Agent based Contextual Navigator, and the implementation of the Probabilistic Outcome Synthesizer.

### 3.3 Counterfactual event planner

Figure 1 presents the Counterfactual Event Planner for evaluating alternative nursing care strategies for oncology patients with cardiovascular risk factors. The Counterfactual Event Planner is structured to address the complexities inherent in the intersection of oncology and cardiovascular care, leveraging advanced computational techniques to simulate and evaluate potential outcomes under various nursing interventions. The three components operate sequentially within a unified counterfactual evaluation framework rather than as independent competing models. The Manifold-Based Decision Model transforms heterogeneous demographic, oncological, cardiovascular, physiological, treatment, and nursing variables into a latent patient representation that preserves clinically relevant structure while reducing noise and redundancy. This representation provides the basis for stable estimation of individualized treatment effects. Using the encoded patient state together with clinical context, the Agent-Based Contextual Navigator explores feasible trajectories under standard acute care and enhanced acute care and identifies the strategy associated with the most favorable predicted outcome. Its outputs therefore directly support policy selection and the evaluation of Policy Value Error. The Probabilistic Outcome Synthesizer assigns strategy-specific outcome distributions to the candidate trajectories, producing estimates of mortality, cardiovascular adverse events, intensive care unit length of stay, and clinical recovery together with their predictive uncertainty. These estimates are used to assess Average Treatment Effect Error, factual outcome prediction error, and outcome calibration. All three modules are trained and evaluated using the same patient cohorts, preprocessing procedures, treatment definitions, outcome labels, data partitions, and evaluation metrics. Their individual contributions are examined through module-removal experiments in which one component is omitted while all other settings remain unchanged, allowing the effect of each module on both task-specific metrics and overall framework performance to be quantified.

**Figure 1:**
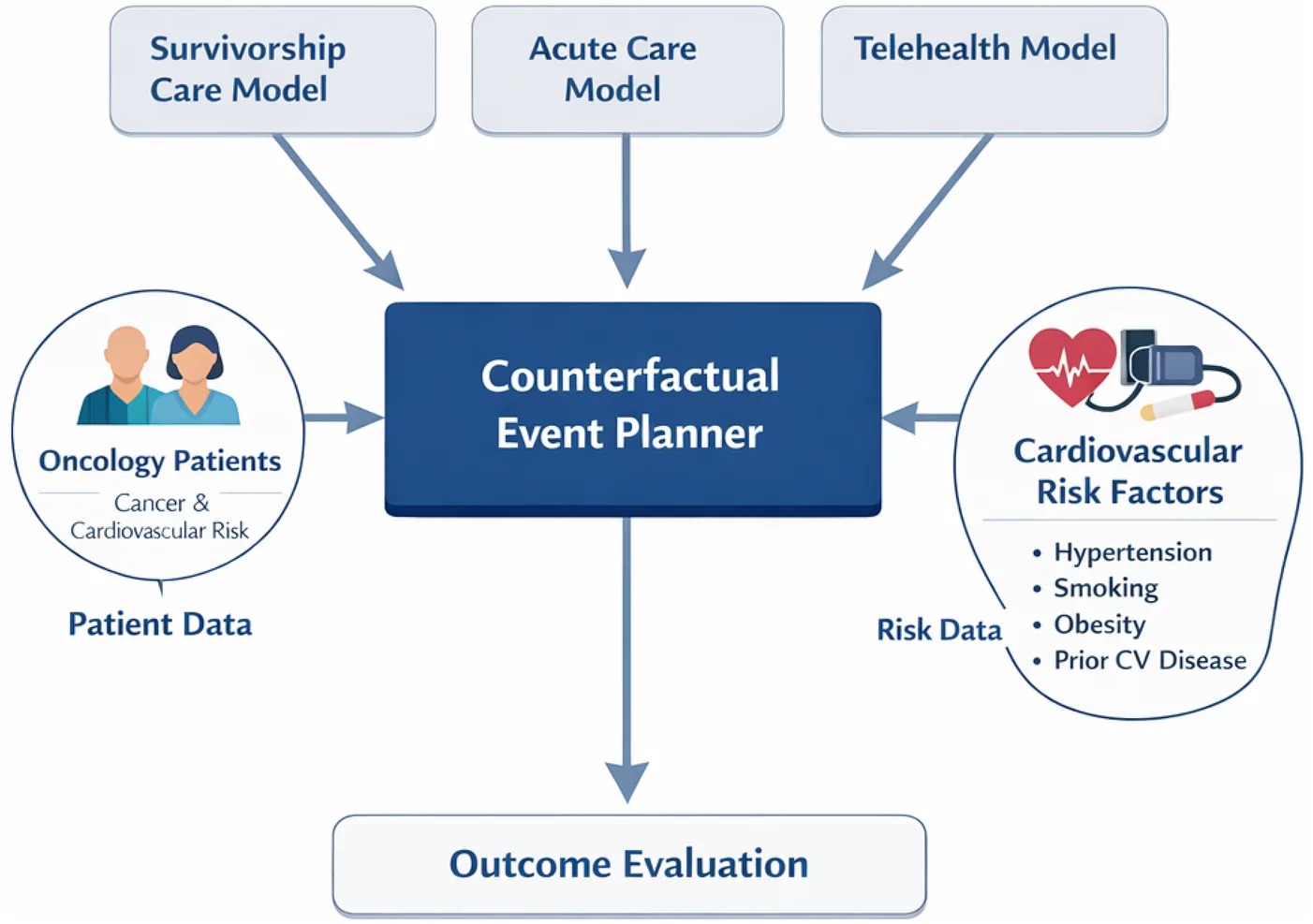
The diagram illustrates the Translational Assessment Framework for Oncology Nursing Care Models, highlighting the central role of the Counterfactual Event Planner. This model integrates patient data and cardiovascular risk factors to evaluate survivorship, acute care, and telehealth models. The framework aims to optimize nursing interventions for oncology patients with cardiovascular risks, facilitating outcome evaluation.

#### 3.3.1 Manifold based decision model

Figure 2 illustrates the three-module architecture of the Counterfactual Event Planner, comprising the Manifold-Based Decision Model, the Agent-Based Contextual Navigator, and the Probabilistic Outcome Synthesizer. Each module plays a critical role in the model’s ability to generate and assess counterfactual scenarios, thereby providing a robust framework for decision making in clinical settings. The Manifold Based Decision Model is responsible for embedding patient data into a high-dimensional manifold, capturing the intricate relationships between various health indicators and risk factors.

**Figure 2:**
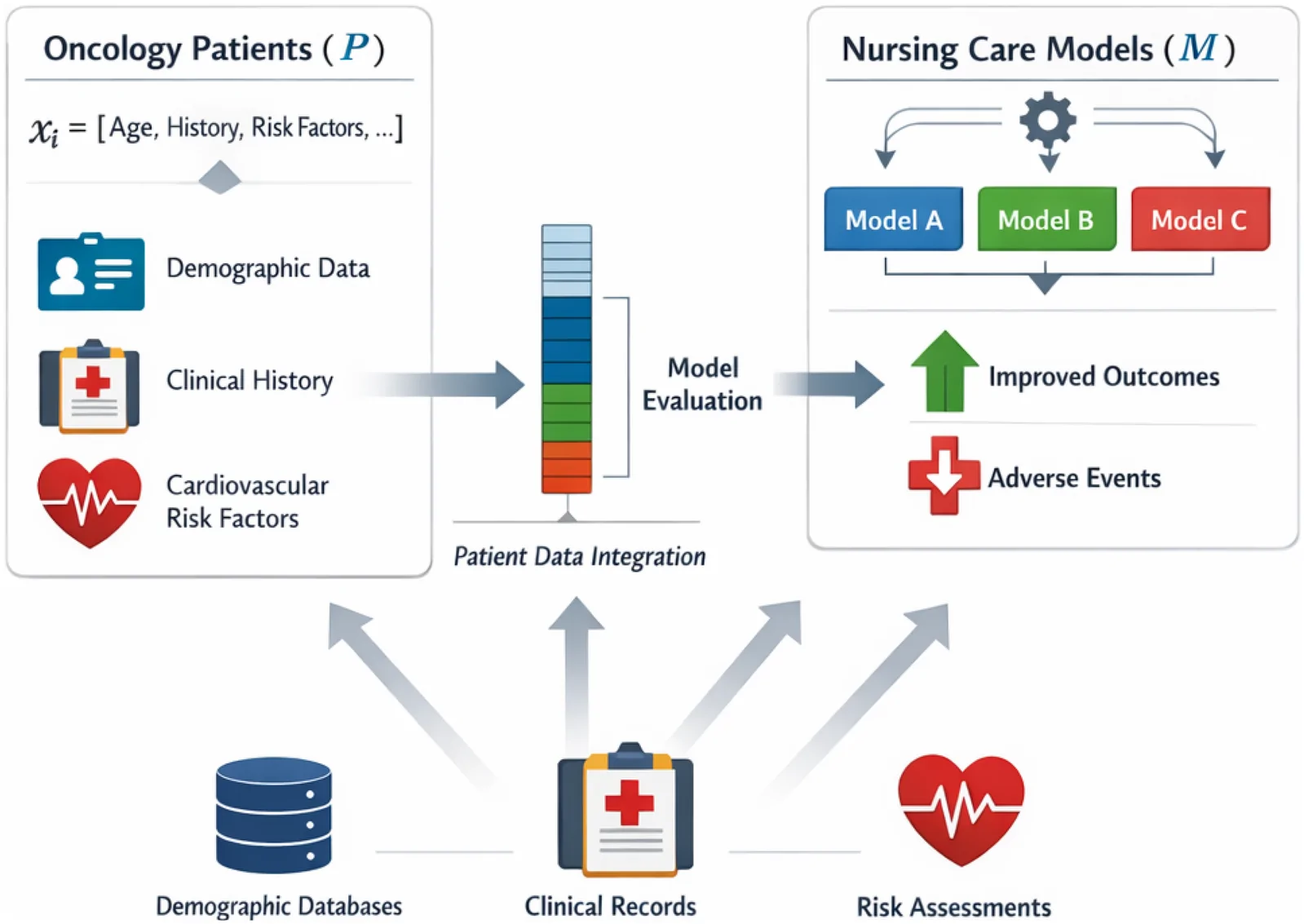
The framework for evaluating nursing care models integrates diverse data sources to assess oncology patients with cardiovascular risk factors. Patient data, including demographic information, clinical history, and cardiovascular risk factors, are synthesized to evaluate different nursing care models. The evaluation aims to optimize patient outcomes by comparing models based on improved outcomes and adverse events.

Let $x\in R^{n}$ represent the vector of patient features, where n denotes the number of features. The encoder maps x to a manifold $M\subset R^{m}$ using a function $f\;:\;R^{n}\to M$, ensuring that the manifold constraints are preserved. The encoding process can be formalized as Equation 5:

$$z=f(x)=\arg\;\min_{z\in M}\|x-g(z){\|}^{2}$$ (5)

where $g\;:\;M\to R^{n}$ is the decoding function that reconstructs the original feature space from the manifold representation. This formulation ensures that the encoded representation z preserves clinically meaningful structures in the original data while simultaneously enforcing smoothness and geometric consistency on the manifold. In practice, the encoder can be implemented using deep representation learning models such as variational autoencoders or manifold regularized neural networks. These models enable the system to capture nonlinear dependencies between physiological indicators such as laboratory measurements, imaging-derived biomarkers, and demographic attributes. By constraining the representation to lie on a learned manifold, the system reduces noise and redundancy in the input space, thereby improving the reliability of downstream decision processes.

For clinical implementation, the encoder is trained on standardized structured variables from the MIMIC IV training cohort. The decoder reconstructs the observed clinical variables under an observed feature mask, which prevents imputed or unavailable measurements from dominating representation learning. This design links the manifold constraint to real oncology and cardiovascular indicators rather than to an unconstrained latent space. The same fitted preprocessing statistics and encoder parameters are then applied unchanged to the validation, internal test, and external eICU cohorts.

#### 3.3.2 Agent based contextual navigation

The Agent based Contextual Navigator utilizes a multi-agent system to explore the manifold space, identifying optimal paths for intervention based on contextual information. Each agent $a_{i}$ operates under a policy $\pi_{i}\;:\;M\times C\to A$, where C represents the contextual information and A denotes the action space. The navigation process is governed by Equation 6:

$$a_{i}=\pi_{i}(z,c)$$ (6)

where c is the context vector derived from patient history and current clinical observations. The contextual information may include temporal medical records, medication history, comorbidities, and environmental or lifestyle factors that influence treatment effectiveness. By incorporating such contextual signals, the policy function allows each agent to adapt its decision strategy dynamically. In the manifold space, navigation can be interpreted as moving from the current patient state $z_{t}$ to a prospective state $z_{t+1}$ that reflects the effect of a potential intervention. This transition can be modeled as Equation 7:

$$z_{t+1}=z_{t}+\Delta (a_{i},z_{t})$$ (7)

where Δ(⋅) represents the state transition function induced by the chosen clinical action. Through collaborative or competitive exploration among multiple agents, the system can efficiently search the manifold for feasible counterfactual trajectories that correspond to different treatment strategies. In this retrospective clinical setting, the navigator is not optimized through online trial and error with patients. The action space is restricted to the predefined clinically observed strategies, namely standard acute care and enhanced acute care. The policy and transition parameters are learned offline from observed care assignments, baseline clinical covariates, temporal nursing records, and outcome labels. This preserves clinical feasibility and avoids unsupported simulated interventions outside the range of observed nursing practice. This multi-agent exploration mechanism allows the framework to evaluate diverse intervention pathways and identify those most likely to improve patient outcomes under varying clinical contexts.

#### 3.3.3 Probabilistic outcome synthesis

The Probabilistic Outcome Synthesizer integrates the outputs from the navigator to simulate potential outcomes under different intervention strategies. This module employs a probabilistic model p(y|a,z) to estimate the likelihood of various health outcomes y. In implementation, the clinical outcome vector contains intensive care unit mortality, in hospital mortality, cardiovascular adverse events, intensive care unit length of stay, and clinical recovery status. The synthesis process estimates the expected potential outcome under a candidate care strategy as Equation 8:

$${\hat{y}}_{i}(a)=E_{\;p_{\theta }(y|a,z_{i})}[y]=\int_{Y}y\;p_{\theta }(y|a,z_{i})\;dy$$ (8)

where $p_{\theta }(y|a,z_{i})$ is parameterized by strategy-specific outcome heads. Bernoulli likelihoods are used for intensive care unit mortality, in hospital mortality, and cardiovascular adverse events. Gaussian likelihoods with learned means and variances are used for intensive care unit length of stay and clinical recovery score.

This probabilistic framework allows for the assessment of uncertainty and variability in patient responses, providing a comprehensive view of potential intervention impacts. The Counterfactual Event Planner offers a sophisticated approach to modeling and evaluating nursing care strategies for oncology patients with cardiovascular risk factors, facilitating informed decision making and personalized care planning.

### 3.4 Counterfactual pacing strategy and Bayesian outcome updating

Figure 3 illustrates how Counterfactual Pacing Strategy and Bayesian Outcome Updating support trajectory simulation and uncertainty-aware outcome estimation within the Counterfactual Event Planner. The strategies, namely Counterfactual Pacing Strategy and Bayesian Outcome Updating, are designed to enhance the predictive accuracy and adaptability of the model, ensuring that it can effectively navigate the multifaceted challenges presented by this unique patient demographic.

**Figure 3:**
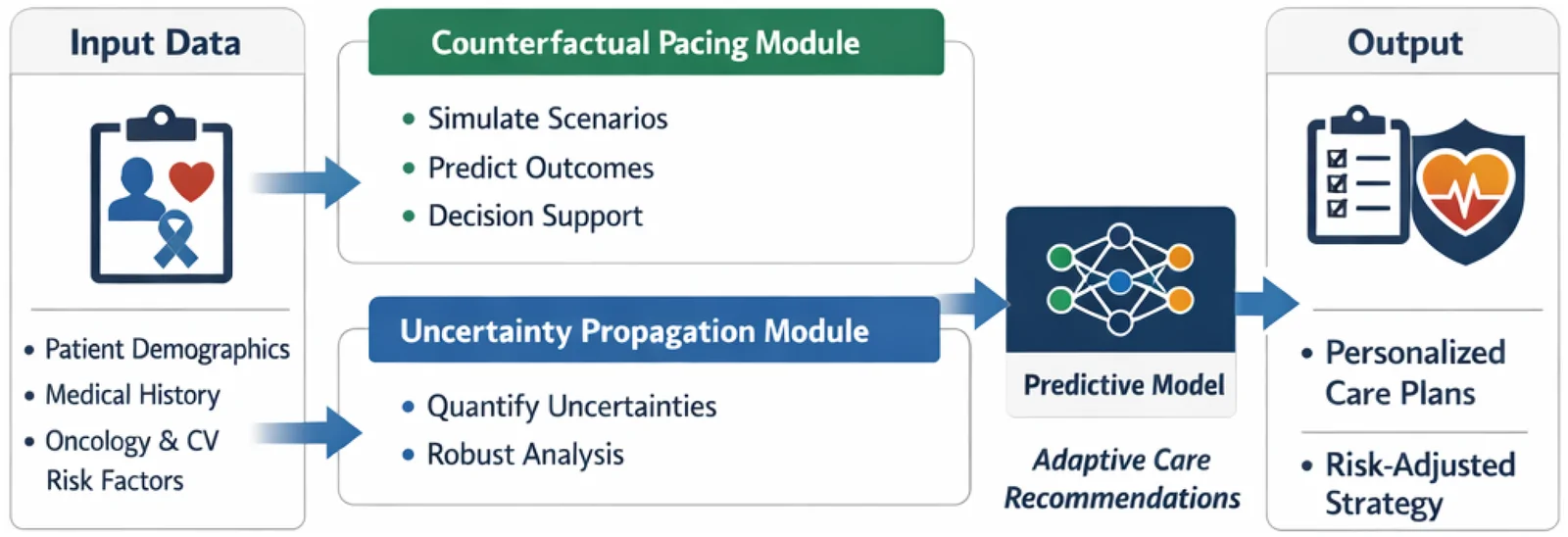
The diagram illustrates the Counterfactual Event Planner framework for oncology patients with cardiovascular risks. Input data, including patient demographics and medical history, feed into the Counterfactual Pacing Strategy and Bayesian Outcome Updating modules. These modules simulate scenarios and quantify uncertainties, respectively, to inform the predictive model. The model generates adaptive care recommendations, resulting in personalized care plans and risk-adjusted strategies.

#### 3.4.1 Counterfactual pacing strategy

Counterfactual Pacing Strategy is a strategic mechanism that enables the model to simulate multiple hypothetical clinical scenarios, allowing healthcare providers to anticipate potential outcomes and proactively adjust treatment strategies. Instead of relying solely on observed trajectories of patient health, the model constructs alternative futures conditioned on different clinical decisions. This capability is particularly valuable in complex medical contexts such as oncology patients with cardiovascular risk factors, where cancer therapies may interact with cardiovascular physiology and lead to uncertain or delayed adverse effects. The ability to forecast these interactions provides clinicians with a decision support framework that balances therapeutic efficacy with cardiovascular safety.

In practical deployment, Counterfactual Pacing Strategy enables the system to explore a spectrum of possible patient trajectories that evolve from the current clinical state. Each trajectory corresponds to a different intervention pathway, such as modifications in drug dosage, scheduling of cardioprotective therapies, or adjustments in monitoring protocols. Through this mechanism, the framework does not merely predict a single outcome but instead evaluates a distribution of plausible futures. This distribution-based reasoning helps clinicians understand the trade offs associated with competing treatment strategies and select interventions that minimize risk while maintaining therapeutic benefit.

The generation of these counterfactual trajectories is facilitated by the integration of the Manifold Based Decision Model and the Agent based Contextual Navigator. The Manifold Based Decision Model transforms heterogeneous clinical data including laboratory results, imaging features, physiological signals, and treatment records into a structured latent representation that respects intrinsic clinical constraints. By embedding patient states into a manifold representation, the model captures nonlinear dependencies among health indicators while reducing noise in the high-dimensional feature space. This structured representation provides a stable foundation for simulating alternative intervention pathways.

Once the patient state has been encoded, the Agent based Contextual Navigator explores the manifold to identify feasible intervention strategies. Multiple agents interact with the latent representation of patient health and contextual information derived from historical medical records, treatment guidelines, and patient specific risk profiles. Each agent proposes potential clinical actions and evaluates their consequences by simulating transitions within the manifold space. Through iterative exploration and policy optimization, the navigator identifies trajectories that correspond to clinically meaningful counterfactual scenarios.

The mathematical formulation of Counterfactual Pacing Strategy can be interpreted as a dynamic system that models the evolution of patient health under varying treatment decisions. Let $x_{t}$ denote the state vector of patient health indicators at time t, which may include biomarkers, cardiovascular measurements, and treatment response variables. Let $u_{t}$ represent the vector of treatment interventions administered at time t. The evolution of the patient state is modeled as Equation 9:

$$x_{t+1}=f(x_{t},u_{t})+\epsilon_{t}$$ (9)

where f(⋅) denotes a nonlinear transition function that captures physiological dynamics and treatment interactions, and $\epsilon_{t}$ represents stochastic perturbations that account for biological variability, measurement noise, and unobserved confounding factors. This formulation allows the system to model patient health as a controlled stochastic process whose trajectory depends on both current health status and intervention decisions.

In the calibrated clinical implementation, $x_{t}$ is the hourly patient state during the first twenty four hours after intensive care admission. It includes vital signs, laboratory variables, cardiovascular measurements, treatment records, nursing observations, and missingness indicators. The intervention vector $u_{t}$ represents the delivered care strategy, physiological monitoring frequency, nursing intensity, and supportive care actions. The transition function is fitted from observed temporal sequences in the MIMIC IV training cohort and is evaluated without refitting on the eICU external cohort.

Counterfactual scenarios are generated by systematically perturbing the intervention vector $u_{t}$ within clinically feasible bounds. By modifying treatment intensity, timing, or combination strategies, the model produces alternative state trajectories that represent hypothetical clinical outcomes. Each simulated trajectory provides insight into how patient health might evolve under different treatment strategies. These simulated outcomes can then be compared to evaluate risk profiles, identify optimal intervention sequences, and support evidence-informed decision making in clinical practice.

Importantly, the pacing mechanism also incorporates temporal consistency constraints to ensure that simulated trajectories remain physiologically plausible. This prevents unrealistic transitions and maintains coherence with known clinical progression patterns. As a result, the framework can generate reliable counterfactual predictions that support long term treatment planning, particularly in high risk patient populations where early intervention and proactive monitoring are critical.

#### 3.4.2 Bayesian outcome updating

Bayesian Outcome Updating complements Counterfactual Pacing Strategy by systematically quantifying the uncertainty associated with each predicted outcome. This strategy is essential for managing the inherent variability in patient responses and treatment efficacy. The Probabilistic Outcome Synthesizer plays a pivotal role in this process, employing advanced probabilistic models to estimate the likelihood of various outcomes under different care scenarios. The synthesis of outcomes is represented by a probability distribution $P(x_{t+1}|x_{t},u_{t})$, which is derived from the convolution of individual probability distributions associated with each health metric and intervention variable.

The Bayesian Outcome Updating strategy is mathematically formalized through the use of Bayesian inference, which updates the probability distribution of patient outcomes as new data becomes available. Let $y_{t}$ denote the observed patient health metrics at time t. The posterior distribution is given by Equation 10:

$$P(x_{t+1}|y_{t})\propto P(y_{t}|x_{t+1})P(x_{t+1}|x_{t},u_{t})$$ (10)

This formulation allows the model to continuously refine its predictions, incorporating real-time data to enhance the accuracy and reliability of its forecasts.

The model is trained and calibrated using clinical data rather than manually assigned reinforcement learning parameters. All imputation values, normalization statistics, exposure thresholds, neural network parameters, transition parameters, posterior parameters, and outcome head parameters are estimated from the MIMIC IV training cohort. Hyperparameters and calibration factors are selected on the MIMIC IV validation cohort. The eICU Collaborative Research Database is used only for external validation and is not used for training, threshold selection, normalization, or calibration.

The complete optimization objective is

$$L=\lambda_{\mathrm{fact}}L_{\mathrm{fact}}+\lambda_{\mathrm{traj}}L_{\mathrm{traj}}+\lambda_{\mathrm{rec}}L_{\mathrm{rec}}+\lambda_{\mathrm{post}}L_{\mathrm{post}},$$

where $L_{\mathrm{fact}}$ is the factual clinical outcome prediction loss, $L_{\mathrm{traj}}$ is the temporal trajectory consistency loss, $L_{\mathrm{rec}}$ is the manifold reconstruction loss, and $L_{\mathrm{post}}$ is the posterior regularization loss. The coefficients are fixed as $\lambda_{\mathrm{fact}}=1.0$, $\lambda_{\mathrm{traj}}=0.5$, $\lambda_{\mathrm{rec}}=0.1$, and $\lambda_{\mathrm{post}}=0.001$. Binary endpoints, including intensive care unit mortality, in hospital mortality, and cardiovascular adverse events, are optimized using binary cross entropy. Continuous endpoints, including intensive care unit length of stay and clinical recovery score, are optimized using Gaussian negative log likelihood.

Calibration is performed on the MIMIC IV validation cohort after training. For binary outcomes, predicted logits are calibrated by temperature scaling. For continuous outcomes, predicted Gaussian variances are calibrated by a validation estimated variance scaling factor. The calibrated parameters are then fixed and applied unchanged to the MIMIC IV internal test cohort and the eICU external test cohort. This procedure connects the mathematical formulation with cardiovascular and oncological clinical data while preventing information leakage from the test cohorts.

In this framework, the strategies of Counterfactual Pacing Strategy and Bayesian Outcome Updating are integral to the functionality of the Counterfactual Event Planner. They provide a robust framework for simulating and evaluating diverse care scenarios, enabling healthcare providers to make informed decisions that optimize patient outcomes in the complex landscape of oncology care with cardiovascular considerations.

### 3.5 Translational phase definition and clinical translation pathway

The translational objective of this study is defined within a staged clinical translation framework that connects computational model development with future bedside application in oncology nursing care. Translational research is commonly described across four phases. T1 translation transforms basic scientific or computational discoveries into candidate clinical tools. T2 translation evaluates the performance, validity, and feasibility of these tools in patient data and controlled clinical research settings. T3 translation examines implementation in routine clinical workflows, including usability, clinical adoption, and integration with care delivery systems. T4 translation evaluates population level impact, long term effectiveness, equity, safety, and health system outcomes after broader dissemination. This study primarily addresses the T1 to T2 translational stage. At the T1 stage, the Counterfactual Event Planner is developed as a computational decision support framework that converts heterogeneous electronic health record variables into patient specific representations and estimates potential outcomes under alternative nursing care strategies. The Manifold Based Decision Model, Agent based Contextual Navigator, Probabilistic Outcome Synthesizer, Counterfactual Pacing Strategy, and Bayesian Outcome Updating are designed to translate clinical observations into individualized counterfactual outcome estimates. At the T2 stage, the framework is evaluated using retrospective critical care records from MIMIC IV and the eICU Collaborative Research Database. Internal validation on MIMIC IV and external validation on eICU are used to examine predictive validity, treatment effect estimation performance, policy quality, and cross-hospital generalization before any prospective clinical deployment. The clinical translation pathway of the proposed framework contains four sequential steps. Structured clinical data are extracted from the first twenty four hours of intensive care admission, including demographic variables, cancer-related information, cardiovascular risk factors, vital signs, laboratory measurements, early treatment records, and nursing records. The model estimates patient specific potential outcomes under standard acute care and enhanced acute care, allowing individualized comparison of nursing strategies. The estimated outcomes are converted into risk-adjusted decision support outputs, including predicted mortality risk, cardiovascular adverse event risk, intensive care unit length of stay, recovery status, and individualized treatment effect. These outputs are intended to support nurse led assessment, interdisciplinary discussion, and care planning after appropriate prospective validation and governance review. The present study does not claim direct T3 or T4 implementation. Instead, it provides methodological evidence required before such implementation can be justified. Future T3 studies should evaluate workflow integration, interpretability for nurses and clinicians, alert burden, human oversight, prospective safety monitoring, and acceptance by multidisciplinary oncology and cardiology care teams. Future T4 studies should examine whether model assisted nursing care improves patient outcomes, reduces cardiovascular complications, decreases unnecessary care variation, supports equitable access to risk-adjusted care, and remains effective across hospitals and patient subgroups. By explicitly positioning the proposed framework within the T1 to T2 phase and outlining the subsequent T3 and T4 requirements, the translational scope of this study is methodologically defined rather than used only as a conceptual description.

To clarify how the computational findings may support practical nursing evaluation, a logic model was developed to connect resources, care activities, immediate outputs, intermediate changes, and long term patient outcomes. As summarized in Table 2, the model outputs are not interpreted as final clinical decisions. They provide structured information that may be translated into nursing assessment priorities, monitoring plans, interdisciplinary communication, and implementation evaluation after prospective validation. The logic model also provides a basis for future implementation evaluation. Model outputs may be incorporated into nursing assessment, handover, cardiovascular risk review, and multidisciplinary care planning. Implementation outcomes should include adoption, fidelity, usability, timeliness, workflow compatibility, alert burden, professional acceptance, patient safety, and equity across clinical subgroups. Process indicators may examine whether risk estimates lead to changes in monitoring frequency, supportive care, escalation, or interdisciplinary communication. Patient outcomes may then be examined to determine whether these process changes are associated with improved recovery and reduced adverse events. This logic model represents a proposed translation pathway and does not imply that prospective clinical implementation has already been completed.

**Table 2:** Logic model for translating computational evaluation into nursing care and implementation assessment.

Logic model element	Application within the proposed framework
Inputs	Electronic health record data, oncology information, cardiovascular risk indicators, nursing observations, monitoring resources, supportive care records, clinical expertise, and institutional protocols.
Activities	Patient risk assessment, estimation of potential outcomes under alternative care strategies, identification of monitoring priorities, review of cardiovascular risk, interdisciplinary discussion, and selection of appropriate nursing actions under clinician supervision.
Direct outputs	Predicted mortality risk, cardiovascular adverse event risk, intensive care unit length of stay, recovery status, individualized treatment effect, and a structured comparison of standard acute care and enhanced acute care.
Intermediate outcomes	Earlier recognition of deterioration, more consistent monitoring, timely escalation of clinical concerns, improved communication among nurses and physicians, greater adherence to care protocols, and more individualized nursing care planning.
long-term outcomes	Reduced mortality and cardiovascular complications, improved clinical recovery, more efficient use of critical care resources, improved continuity of care, and safer and more equitable oncology nursing services.

### 3.6 Mixed methods study design

This study adopted a quantitatively dominant mixed methods design that integrated retrospective electronic health record analysis with clinical interpretive analysis. This design was used to connect computational model development with clinically meaningful nursing care assessment. The quantitative strand provided statistical estimation, counterfactual prediction, and external validation, while the qualitative clinical strand supported problem formulation, variable interpretation, care strategy definition, and translation of model outputs into nursing care contexts. The detailed mixed methods components and integration strategy are summarized in Table 3. The quantitative strand was based on structured electronic health record data from MIMIC IV and the eICU Collaborative Research Database. MIMIC IV was used for model training, validation, and internal testing, and the eICU Collaborative Research Database was used for external validation. The quantitative analysis included cohort construction, feature extraction, treatment assignment definition, outcome label construction, model training, calibration, comparative evaluation, semi-synthetic counterfactual validation, and ablation analysis. The main clinical inputs included demographic characteristics, oncology related diagnosis information, cardiovascular risk factors, vital signs, laboratory measurements, early treatment records, nursing observations, and monitoring variables. The main outcomes included intensive care unit mortality, in hospital mortality, cardiovascular adverse events, intensive care unit length of stay, and clinical recovery status. The qualitative clinical strand was used to ensure that the computational task corresponded to oncology nursing practice and cardiovascular risk management. This strand included structured interpretation of the clinical meaning of input variables, care strategy definitions, and outcome measures. Nursing care strategies were interpreted according to early nursing intensity, physiological monitoring frequency, and supportive care activities. Cardiovascular risk variables were interpreted according to documented comorbidities, abnormal physiological states, cardiovascular diagnosis records, and clinically relevant laboratory indicators. Oncology variables were interpreted according to cancer diagnosis records, cancer-related admission information, and severity indicators available in structured clinical data. The integration of the quantitative and qualitative strands occurred at three stages. Design integration was performed during cohort construction and feature selection, where clinical nursing concepts were mapped to structured electronic health record variables. Analytical integration was performed during treatment and outcome definition, where standard acute care and enhanced acute care were operationalized using measurable nursing and monitoring indicators. Interpretation integration was performed during result analysis, where statistical performance, treatment effect estimates, policy value, and model uncertainty were interpreted in relation to nursing decision support and translational feasibility. The mixed methods structure links electronic health record based quantitative modeling with clinical interpretation across the design, analytical, and interpretation stages. This design clarifies that the study is not limited to algorithmic prediction. Instead, quantitative electronic health record analysis and qualitative clinical interpretation are integrated to support a clinically grounded assessment of nursing care models for oncology patients with cardiovascular risk factors. The mixed methods structure therefore strengthens the methodological transparency of the study and explains how computational evidence is connected to nursing care interpretation and future clinical translation.

**Table 3:** Mixed methods design and integration strategy.

Component	Data or evidence source	Purpose	Output
Quantitative electronic health record analysis	Structured records from MIMIC IV and the eICU Collaborative Research Database	Estimate factual and counterfactual outcomes, evaluate care strategy effects, and test cross-hospital generalization	Model performance, treatment effect estimates, policy value, calibration results, and robustness results
Clinical interpretive analysis	Oncology nursing concepts, cardiovascular risk management principles, and clinically available structured variables	Define clinically meaningful variables, care strategies, and outcome interpretations	Mapping between electronic health record variables, nursing care concepts, cardiovascular risk indicators, and outcome labels
Design integration	Cohort criteria, feature extraction rules, and clinical inclusion logic	Align computational data construction with the target nursing care problem	Clinically grounded oncology cohort with cardiovascular risk factors
Analytical integration	Treatment assignment rules, outcome labels, and model outputs	Connect care strategy definitions with counterfactual modeling and outcome estimation	Operational definitions of standard acute care, enhanced acute care, mortality, cardiovascular adverse events, length of stay, and recovery status
Interpretation integration	Model comparison results, external validation results, semi-synthetic validation, and ablation analysis	Interpret statistical findings in relation to nursing care assessment and translational decision support	Clinically interpretable evidence for individualized nursing care strategy evaluation

The interpretation of the nursing care system was further guided by the structure, process, and outcome framework. Structural factors refer to the organizational and material conditions under which care is delivered, including nurse staffing, professional experience, availability of monitoring equipment, information system capacity, access to oncology and cardiology services, institutional protocols, and hospital resource levels. These factors may influence process factors by determining whether frequent physiological monitoring, timely nursing assessment, supportive care, interdisciplinary communication, and escalation of clinical concerns can be delivered consistently. The process factors represented in the present study include nursing intensity, physiological monitoring frequency, supportive care activities, and the observed acute care strategy. These care processes may subsequently affect patient outcomes through earlier recognition of deterioration, more timely clinical intervention, improved coordination, and better management of cardiovascular complications. Intensive care unit mortality, in hospital mortality, cardiovascular adverse events, intensive care unit length of stay, and clinical recovery status were interpreted as downstream outcomes arising from the interaction between structural conditions and care processes. Cross hospital validation using the eICU Collaborative Research Database provides an initial assessment of model transportability across heterogeneous care environments, but it does not establish the independent causal effects of specific structural factors. Future prospective studies should therefore collect explicit hospital and unit-level variables and evaluate whether structural conditions modify the relationship between nursing care processes and patient outcomes.

## 4 Experiments

### 4.1 Task definition

The experimental task is formulated as individual treatment effect estimation for oncology patients admitted to intensive care with cardiovascular risk factors. The input X consists of the clinical state recorded during the early stage of acute admission, including demographic characteristics, cancer-related information, previous disease history, cardiovascular risk factors, vital signs, laboratory measurements, early treatment records, and nursing records. The intervention variable A represents the acute care strategy received by each patient. Acute care strategies are defined according to nursing intensity, physiological monitoring frequency, and supportive care measures delivered during the early admission period. The output Y contains the observed clinical outcomes associated with each strategy. The primary outcome is mortality, represented by intensive care unit mortality and in hospital mortality. The secondary outcomes include cardiovascular adverse events, intensive care unit length of stay, and clinical recovery status. The outcome indicators were selected according to their clinical relevance to oncology nursing and critical care, their capacity to represent complementary dimensions of care effectiveness, and their consistent availability in both MIMIC IV and the eICU Collaborative Research Database. Intensive care unit mortality reflects acute deterioration during a period in which continuous nursing assessment, physiological monitoring, early warning recognition, and timely escalation of care are particularly important. In hospital mortality provides a broader measure of survival across the complete hospitalization and captures adverse outcomes occurring after transfer from intensive care. Cardiovascular adverse events were included because cardiovascular complication prevention and early recognition are central care priorities for oncology patients with cardiovascular risk factors. Intensive care unit length of stay reflects illness burden, recovery speed, care intensity, and critical care resource utilization. Clinical recovery status complements mortality outcomes by incorporating physiological improvement, changes in organ support requirements, and discharge disposition. These indicators provide a multidimensional assessment of patient safety, cardiovascular complications, resource use, and clinical recovery, while remaining objectively extractable from structured critical care records.

The learning objective is to estimate the potential outcome of each patient under every predefined acute care strategy and calculate the difference between these potential outcomes. The task therefore follows a regression paradigm within observational causal inference, because the model predicts patient specific potential outcomes and continuous treatment effect values rather than directly reproducing the observed treatment assignment. The supervision signal is obtained from system statistics recorded in the electronic health records of MIMIC IV and the eICU Collaborative Research Database. Each patient record provides an observed acute care strategy, baseline clinical covariates, and corresponding clinical outcomes generated during routine intensive care practice. Mortality labels are derived from recorded discharge status and death information. Cardiovascular adverse event labels are extracted from diagnosis records, clinical event records, and treatment documentation. Intensive care unit length of stay is calculated from admission and discharge timestamps. Clinical recovery status is determined from recorded physiological improvement, organ support requirements, and discharge disposition. These supervision signals are generated from structured hospital information systems rather than manual annotation. Because only the outcome under the observed strategy is available for each patient, the outcomes under unobserved strategies remain counterfactual. Training therefore combines supervised outcome prediction with causal representation learning to infer missing potential outcomes while controlling for differences in baseline patient characteristics and treatment assignment.

### 4.2 Dataset and data preprocessing

#### 4.2.1 Datasets

The experiments use two public critical care electronic health record databases. MIMIC IV contains 299,712 patients, 431,231 hospital admissions, and 73,181 intensive care unit stays collected from a tertiary academic medical center, and it is used for model development, training, and internal validation. The eICU Collaborative Research Database contains 200,859 intensive care unit encounters from 139,367 patients treated at 208 hospitals across the United States, and it is used exclusively for external validation. Labels are derived from structured system statistics and clinical records without manual annotation. Intensive care unit mortality and in hospital mortality are determined from recorded death indicators and discharge status. Cardiovascular adverse events are identified from diagnosis codes and documented clinical events. Intensive care unit length of stay is calculated from admission and discharge timestamps, while clinical recovery status is determined from physiological improvement, organ support withdrawal, and discharge disposition. Acute care strategy labels are constructed from nursing intensity, physiological monitoring frequency, and supportive care measures recorded during the early admission period. Adult oncology patients aged at least 18 years are retained when a cancer diagnosis, an intensive care unit admission, cardiovascular risk information, and complete outcome records are available. Encounters lasting less than 24 h are removed because they provide insufficient early intervention information. Records with missing intervention assignments, missing mortality status, duplicated admissions, or implausible physiological values are excluded. Continuous variables are winsorized at the first and ninety ninth percentiles, missing numerical values are imputed using training set medians, categorical variables are assigned an explicit missing category, and all numerical features are standardized using training set statistics. MIMIC IV is divided at the patient level into training, validation, and internal test sets using an 8:1:1 random split, ensuring that all admissions from the same patient remain in one subset. The complete eICU cohort is kept separate as a cross-hospital external test set, preventing patient and hospital information from entering model development.

The experimental datasets consisted of MIMIC IV (36) and the eICU Collaborative Research Database (37). MIMIC IV is a public critical care electronic health record resource collected from a tertiary academic medical center, and the eICU Collaborative Research Database is a public multi center critical care database collected from hospitals across the United States. The oncology cohort was constructed from these two databases according to predefined clinical criteria rather than from an independent Oncology Patient Care Models Dataset. Eligible records were adult intensive care encounters with cancer diagnosis information, cardiovascular risk information, early clinical and nursing variables, and complete outcome records. The database level characteristics, experimental roles, cohort construction rules, label definitions, and supporting references are summarized in Table 4.

**Table 4:** Dataset information and literature support.

Dataset	Public source and scale	Experimental use	Cohort construction and labels	Literature
MIMIC IV	A public critical care and hospital electronic health record database collected from a tertiary academic medical center. The database contains 299,712 patients, 431,231 hospital admissions, and 73,181 intensive care unit stays.	Model development, training, validation, and internal testing. The patient level training, validation, and internal test split follows an 8:1:1 ratio.	Adult oncology intensive care encounters are selected when cancer diagnosis records, intensive care admission records, cardiovascular risk information, and complete outcome records are available. Encounters shorter than twenty four hours, duplicated admissions, missing intervention assignments, missing mortality status, and implausible physiological values are excluded. Mortality labels are derived from death indicators and discharge status. Cardiovascular adverse events are identified from diagnosis records and documented clinical events. Intensive care unit length of stay is calculated from admission and discharge timestamps.	(36)
eICU Collaborative Research Database	A public multi center critical care electronic health record database collected from hospitals across the United States. The database contains 200,859 intensive care unit encounters from 139,367 patients treated at 208 hospitals.	External cross-hospital validation. The complete eligible eICU cohort is reserved for testing without retraining, threshold adjustment, or cohort specific hyperparameter optimization.	The same cohort definition, exclusion rules, feature construction procedure, treatment definition, and outcome label extraction strategy are applied to eICU. This design evaluates generalization across hospitals, patient populations, care practices, and measurement patterns.	(37)

#### 4.2.2 Data preprocessing

Data preprocessing is performed consistently for MIMIC IV and the eICU Collaborative Research Database. Duplicate intensive care unit encounters are removed according to patient identifier, hospital admission identifier, intensive care unit stay identifier, and admission timestamp. Repeated measurements recorded at the same timestamp are aggregated using the median for continuous variables and the most frequent value for categorical variables. Variables with more than 40% missing observations in the training cohort are excluded. Remaining continuous variables are imputed using the median calculated from the MIMIC IV training set, while categorical variables are assigned a dedicated unknown category. Missingness indicators are retained for laboratory measurements, vital signs, treatment records, and nursing observations. Physiological values outside clinically valid ranges are treated as missing, including heart rate below 20 or above 250 beats per minute, systolic blood pressure below 40 or above 300 mmHg, diastolic blood pressure below 20 or above 200 mmHg, respiratory rate below 4 or above 80 breaths per minute, body temperature below 30 or above 43 degrees Celsius, and oxygen saturation below 50% or above 100%. Continuous laboratory variables are further clipped at the first and ninety ninth percentiles of the training distribution. All numerical features are standardized using training set means and standard deviations, and categorical variables are converted into integer encoded categories. Clinical events are aligned to the intensive care unit admission time, and only information recorded during the first 24 h is retained. Sequential measurements are divided into hourly intervals, with the median value used within each interval and forward filling restricted to six consecutive hours. Patient identity is aligned across admissions, diagnoses, laboratory records, medication records, procedures, vital signs, and nursing tables through database specific patient and encounter identifiers. Acute care strategy exposure is constructed from nursing intensity, monitoring frequency, and supportive care delivered within the same 24 h window. No synthetic augmentation or external feature extractor is used because all inputs are structured electronic health record variables.

### 4.3 Evaluation metrics and baseline

#### 4.3.1 Metrics definition

Four primary metrics evaluate individual effect estimation, population effect estimation, policy quality, and outcome prediction. Precision in Estimation of Heterogeneous Effect measures the root mean squared difference between the estimated individual treatment effect ${\hat{\tau }}_{i}$ and the cross fitted doubly robust reference effect ${\tilde{\tau }}_{i}$: $\mathrm{PEHE}=\sqrt{\frac{1}{N}\sum_{i=1}^{N}({\hat{\tau }}_{i}-{\tilde{\tau }}_{i}{)}^{2}}$. Average Treatment Effect Error measures the absolute deviation between the estimated and reference population effects: $\mathrm{ATE}\;\mathrm{Error}=\left|\frac{1}{N}\sum_{i=1}^{N}{\hat{\tau }}_{i}-\frac{1}{N}\sum_{i=1}^{N}{\tilde{\tau }}_{i}\right|$. Policy Value Error evaluates whether the estimated treatment effects support correct acute care decisions. The learned policy is defined as $\hat{\pi }(x_{i})=\arg\;\max_{a}{\hat{Y}}_{i}(a)$, and its error is $\left|\tilde{V}(\hat{\pi })-\tilde{V}(\pi^{⋆})\right|$, where survival is used as the clinical utility. Outcome Prediction Error evaluates factual outcome estimation through $\mathrm{RMSE}=\sqrt{\frac{1}{N}\sum_{i=1}^{N}(Y_{i}-{\hat{Y}}_{i}{)}^{2}}$, while mean absolute error is reported as a complementary value. Lower values indicate better performance for all four metrics. Computational efficiency is measured using the total number of trainable parameters and the floating point operations required for one patient level forward pass with batch size one.

#### 4.3.2 Evaluation protocol

The acute care exposure is represented as a binary treatment variable. Enhanced acute care is assigned when the composite score of early nursing intensity, physiological monitoring frequency, and supportive care reaches the median calculated from the MIMIC IV training cohort. Standard acute care is assigned below this threshold. Every model receives the same first 24 h clinical variables and predicts the potential outcomes under both strategies. Policy evaluation uses Top K with K=1, meaning that the strategy with the highest predicted survival probability is selected for each patient. MIMIC IV is divided at the patient level into training, validation, and internal test sets with an 8:1:1 random ratio. All admissions belonging to the same patient remain in one subset. The validation set is used for hyperparameter selection and early stopping, while the internal test set is accessed only after model development. The complete eICU cohort is reserved for cross-hospital external evaluation. Seen evaluation refers to previously unobserved MIMIC IV patients drawn from the development institution, whereas unseen evaluation refers to patients and hospitals contained only in eICU. Five fold cross fitting produces doubly robust reference effects for PEHE, ATE Error, and Policy Value Error without using test outcomes during model fitting. All experiments follow an offline protocol based on retrospective records. Temporal online deployment, intersection over union evaluation, and keypoint accuracy thresholds are not used because the task concerns structured clinical causal estimation rather than detection, segmentation, or pose estimation.

#### 4.3.3 Statistical settings

Each model is independently trained and evaluated ten times using ten distinct random seeds. The patient level dataset split remains fixed across all methods to ensure that performance differences arise from model estimation rather than changes in cohort composition. Random seeds control parameter initialization, training batch order, dropout masks, causal forest sampling, propensity model fitting, and cross fitting fold assignment. The same seed index is applied to every competing method, creating paired experimental observations for statistical comparison. Results for PEHE, ATE Error, Policy Value Error, outcome prediction error, parameter count, and floating point operations are reported as the arithmetic mean plus or minus the standard deviation across the ten runs. Internal validation results are calculated on the MIMIC IV test cohort, and external validation results are calculated independently on the complete eligible eICU cohort. Statistical significance between the proposed method and each baseline is assessed using a two sided paired Student’s t test. Pairing is performed by random seed so that the result from one method is compared with the result from the competing method under the same experimental conditions. A difference is considered statistically significant when p<0.05. The significance test is conducted separately for every primary metric and evaluation cohort. Model selection is completed using only the MIMIC IV validation set, and no test result is used to modify preprocessing rules, treatment construction, hyperparameters, stopping criteria, or decision thresholds.

#### 4.3.4 Baseline

Six baselines provide comparisons across traditional statistics, conventional causal inference, deep learning, causal representation learning, state of the art causal estimation, and lightweight ensemble learning. Logistic Regression is implemented as an outcome regression model using patient covariates, treatment assignment, and covariate treatment interaction terms. It estimates both potential outcomes by setting the treatment indicator to zero and one during inference. Propensity Score Matching estimates treatment probability through logistic regression and applies one to one nearest neighbor matching without replacement using a caliper equal to 0.2 standard deviations of the logit propensity score. Multi Layer Perceptron serves as the standard deep learning baseline and predicts clinical outcomes from the concatenation of the patient representation and treatment indicator. TARNet learns a shared patient representation followed by two treatment specific outcome heads, separating representation learning from potential outcome estimation. DragonNet extends this structure with an additional propensity prediction head and targeted regularization, jointly optimizing treatment assignment and outcome estimation. Causal Forest represents the lightweight baseline and uses an ensemble of honest causal trees to estimate heterogeneous treatment effects through recursive partitioning of the clinical feature space. Every baseline uses identical patient cohorts, preprocessing outputs, treatment definitions, outcome labels, training splits, and evaluation metrics. Hyperparameters are selected exclusively on the MIMIC IV validation set, and the selected configuration is transferred unchanged to internal and external testing.

### 4.4 Implementation details

All experiments are executed on a workstation equipped with one Intel Xeon Gold 6226R CPU, one NVIDIA GeForce RTX 3090 GPU with 24 GB video memory, 256 GB system RAM, and Ubuntu 22.04 LTS. The implementation uses Python 3.10.13 and PyTorch 2.1.2 with CUDA 12.1 and cuDNN 8.9.7. Data processing is performed with NumPy 1.26.4, pandas 2.1.4, and scikit learn 1.4.0. Causal Forest is implemented with EconML 0.15.0, and parameter counts and floating point operations are calculated with fvcore 0.1.5. The proposed model is trained for a maximum of 200 epochs with a batch size of 256. AdamW is used as the optimizer with an initial learning rate of $1\times 10^{-3}$ and a weight decay of $1\times 10^{-5}$. A ReduceLROnPlateau scheduler monitors the total validation loss, reduces the learning rate by a factor of 0.5 after five epochs without improvement, and sets the minimum learning rate to $1\times 10^{-6}$. Gradient norms are clipped to 5.0 to stabilize optimization. Early stopping is activated when the validation loss does not improve for 20 consecutive epochs, and the checkpoint with the lowest validation loss is retained. Ten independent runs use the fixed random seeds {0,1,2,3,4,5,6,7,8,9}. Each seed controls patient shuffling, parameter initialization, dropout masks, mini batch ordering, cross fitting partitions, and tree sampling. Binary outcomes are optimized using binary cross entropy, while continuous outcomes are optimized using mean squared error. The complete training objective combines factual outcome prediction, manifold reconstruction, trajectory consistency, and Bayesian posterior regularization. All preprocessing statistics are fitted on the MIMIC IV training split and reused unchanged for validation, internal testing, and external testing.

The model receives the first 24 h of each intensive care unit stay, represented as 24 aligned hourly bins. Continuous measurements within each bin are aggregated using the median, categorical events are converted into fixed indices, and missingness indicators are concatenated with the observed clinical variables. Each categorical variable is mapped to a 16 dimensional embedding, after which all hourly features are projected into a 128 dimensional patient representation. The Manifold Based Decision Model employs a three layer encoder with hidden dimensions of 256, 128, and 64, GELU activation functions, layer normalization, and a dropout rate of 0.1. The latent manifold dimension is fixed at 64. A symmetric decoder with hidden dimensions of 64, 128, and 256 reconstructs the standardized clinical input, and the reconstruction loss coefficient is set to 0.1. The Agent based Contextual Navigator contains four agents. Each agent employs a two layer policy network with hidden dimensions of 128 and 64 and selects one of the two predefined acute care strategies. The latent transition network contains two hidden layers of 128 units and recursively updates the patient state across the 24 hourly steps. The Probabilistic Outcome Synthesizer contains two shared hidden layers with dimensions of 128 and 64, followed by strategy-specific outcome heads. Intensive care unit mortality, in hospital mortality, and cardiovascular adverse events are modeled using Bernoulli likelihoods. Intensive care unit length of stay and clinical recovery scores are modeled using Gaussian likelihoods with learned means and log variances. Counterfactual Pacing generates one patient trajectory under each acute care strategy from the same baseline state. Bayesian Outcome Updating maintains a diagonal Gaussian posterior over the latent health state and performs one posterior update after every hourly observation. Loss coefficients are fixed at 1.0 for factual outcome prediction, 0.5 for trajectory consistency, 0.1 for manifold reconstruction, and $1\times 10^{-3}$ for posterior regularization. The individualized treatment effect is calculated as the difference between the two predicted potential outcomes. All proposed and baseline methods are trained and evaluated using the same patient level data splits, preprocessing pipeline, treatment definition, outcome labels, hardware environment, and statistical protocol. Hyperparameters for every method are selected only on the MIMIC IV validation set using an identical search budget of 30 configurations. The selected configuration is fixed before internal testing and transferred unchanged to the complete eICU external test cohort. Every method is trained using the same ten random seeds. Neither test cohort is used for model selection, threshold adjustment, early stopping, feature processing, or hyperparameter tuning.

To improve the transparency and reproducibility of the empirical evaluation, Table 5 presents a consolidated description of the experimental design and implementation settings. It specifies the analytical sample allocation, model inputs and outputs, training procedure, principal parameter settings, repeated experiments, computing environment, and evaluation criteria. This summary also clarifies the separation of model development, internal testing, and cross-hospital external validation under a consistent experimental protocol.

**Table 5:** Summary of the experimental design.

Component	Specification
Data allocation	The eligible MIMIC IV cohort contained 8,640 intensive care encounters, including 6,912 for training, 864 for validation, and 864 for internal testing according to an 8:1:1 patient level split. The eligible eICU cohort contained 9,760 encounters and was used exclusively for external validation.
Input, intervention, and output	Inputs included demographic, oncology, cardiovascular, physiological, laboratory, treatment, and nursing variables recorded during the first 24 h of intensive care admission. The intervention represented standard acute care or enhanced acute care. Outputs included mortality, cardiovascular adverse events, intensive care unit length of stay, clinical recovery status, and individual treatment effects.
Training settings	Preprocessing and model training were performed using the MIMIC IV training set. The validation set supported parameter selection, calibration, and early stopping. Training used AdamW for a maximum of 200 epochs with a batch size of 256, an initial learning rate of $1\times 10^{-3}$, a weight decay of $1\times 10^{-5}$, a latent dimension of 64, a dropout rate of 0.1, and four contextual agents.
Repeated experiments	Each method was trained and evaluated ten times using random seeds from 0 to 9. Results were reported as the mean and standard deviation. Statistical comparisons used a two sided paired Student’s t test with p<0.05.
Computing environment	Experiments were conducted using an Intel Xeon Gold 6226R processor, an NVIDIA GeForce RTX 3090 graphics processor with 24 GB memory, 256 GB system memory, Ubuntu 22.04 LTS, Python 3.10.13, and PyTorch 2.1.2.
Evaluation criteria	Evaluation included PEHE, Average Treatment Effect Error, Policy Value Error, root mean squared error, mean absolute error, parameter count, and floating point operations. External validation, semi-synthetic validation, component removal analysis, sensitivity analysis, and missing data robustness analysis were also conducted.

### 4.5 Results and discussion

#### 4.5.1 Comparative experiments

Comparative experiments evaluate the proposed Counterfactual Event Planner against six representative baselines: Logistic Regression, Propensity Score Matching, Multi Layer Perceptron, TARNet, DragonNet, and Causal Forest. These methods cover traditional statistical modeling, conventional causal inference, standard deep learning, causal representation learning, state of the art treatment effect estimation, and lightweight tree based learning. Performance is measured using PEHE, ATE Error, Policy Value Error, RMSE, and MAE. PEHE assesses individual treatment effect estimation, ATE Error measures population level effect estimation, Policy Value Error evaluates the quality of acute care strategy selection, and RMSE and MAE quantify factual outcome prediction accuracy. Computational efficiency is assessed through the number of model parameters and floating point operations required for patient level inference. MIMIC IV is used for model development and internal testing, while the eICU Collaborative Research Database is reserved for cross-hospital external testing. All methods use the same patient cohorts, first 24 h observation window, preprocessing pipeline, treatment definition, outcome labels, and evaluation metrics. Hyperparameters are selected only on the MIMIC IV validation set under the same search budget. Each experiment is repeated with ten identical random seeds across methods, and results are reported as mean ± standard deviation. Statistical significance is evaluated using a two sided paired Student’s t test with p<0.05.

Table 6 summarizes the internal evaluation results for previously unseen patients in the MIMIC-IV cohort. The Counterfactual Event Planner achieved the lowest error across all five metrics, indicating consistent improvements in individualized effect estimation, population-level effect estimation, policy selection, and factual outcome prediction. Its PEHE was 0.099, compared with 0.111 for DragonNet, the strongest-performing baseline, representing a relative reduction of 10.8%. The ATE Error reached 0.018 rather than DragonNet’s 0.022, while the corresponding Policy Value Error values were 0.023 and 0.027, respectively. These differences amount to relative reductions of 18.2% in ATE Error and 14.8% in Policy Value Error. The proposed method also produced lower RMSE and MAE values of 0.185 and 0.134, compared with 0.192 and 0.139 for DragonNet. Collectively, these findings indicate that the estimated patient-specific effects provide more accurate outcome estimates and more reliable support for individualized acute care strategy selection. The observed gains may reflect the contribution of the Manifold-Based Decision Model in preserving clinically meaningful nonlinear structure while reducing noise and redundancy in heterogeneous electronic health records.

**Table 6:** Comparative results on the MIMIC IV internal test cohort.

Method	PEHE ↓	ATE Error ↓	Policy Value Error ↓	RMSE ↓	MAE ↓
Logistic regression	0.146±0.004	0.036±0.003	0.041±0.003	0.222±0.003	0.163±0.002
Propensity score matching	0.153±0.005	0.041±0.004	0.046±0.004	0.229±0.004	0.170±0.003
Multi layer perceptron	0.132±0.004	0.031±0.003	0.036±0.003	0.207±0.003	0.151±0.002
TARNet	0.119±0.003	0.026±0.002	0.031±0.002	0.198±0.002	0.144±0.002
DragonNet	0.111±0.003	0.022±0.002	0.027±0.002	0.192±0.002	0.139±0.002
Causal forest	0.124±0.004	0.028±0.003	0.033±0.003	0.203±0.003	0.148±0.002
Counterfactual event planner	0.099±0.002	0.018±0.001	0.023±0.001	0.185±0.002	0.134±0.001

Each entry reports mean ± standard deviation over ten independent runs. Lower values indicate better performance.

The Agent based Contextual Navigator incorporates temporal history, medication information, and cardiovascular comorbidity context to explore clinically feasible intervention trajectories rather than relying on a static patient representation. Compared with the standard Multi Layer Perceptron, the proposed method reduces PEHE by 25.0% and Policy Value Error by 36.1%, demonstrating the benefit of explicit counterfactual modeling beyond conventional outcome prediction. The Counterfactual Pacing Strategy further improves individualized effect estimation by simulating both acute care trajectories from a shared baseline state. Meanwhile, the Probabilistic Outcome Synthesizer and Bayesian Outcome Updating continuously represent and revise predictive uncertainty using newly observed clinical information, reducing RMSE from 0.192 to 0.185 and MAE from 0.139 to 0.134 relative to DragonNet. The proposed model also exhibits the smallest or jointly smallest standard deviation for all metrics, suggesting improved optimization stability across random seeds. The results indicate that integrating manifold representation, context-aware trajectory exploration, counterfactual simulation, and uncertainty-aware outcome updating provides a consistent advantage over statistical, tree-based, and neural causal estimators.

Table 7 presents the cross-hospital evaluation on the eICU Collaborative Research Database without retraining, threshold adjustment, or cohort-specific hyperparameter optimization. Although all methods exhibit higher errors than on the MIMIC IV internal cohort, their overall ranking remains stable, indicating that the external results primarily reflect institutional distribution shift rather than unstable model behavior. The proposed Counterfactual Event Planner achieves the lowest mean error for all evaluation metrics. Compared with DragonNet, the strongest competing neural causal estimator, our method reduces PEHE from 0.128 to 0.113, corresponding to an improvement of 11.7%. ATE Error decreases from 0.027 to 0.022, while Policy Value Error is reduced from 0.032 to 0.027, yielding relative improvements of 18.5% and 15.6%, respectively. These results indicate that the proposed framework preserves both individualized treatment effect accuracy and clinically relevant acute care selection under changes in patient composition, clinical practice, and measurement patterns. The improved external performance can be attributed to the Manifold Based Decision Model, which constrains heterogeneous clinical observations to a structured latent space and reduces sensitivity to institution-specific noise. Furthermore, the Agent based Contextual Navigator integrates temporal history, medication information, and cardiovascular comorbidity context when exploring feasible intervention trajectories, thereby improving transportability beyond static representation learning. Relative to the Multi Layer Perceptron, the proposed model reduces external PEHE by 25.7% and Policy Value Error by 37.2%, demonstrating that the gain is not solely explained by increased nonlinear modeling capacity. The Counterfactual Pacing Strategy contributes by evaluating both acute care trajectories from a shared patient state, whereas Bayesian Outcome Updating continuously revises the latent health distribution as clinical evidence becomes available. Consequently, RMSE decreases from 0.207 to 0.199 and MAE from 0.151 to 0.145 compared with DragonNet. The proposed method maintains standard deviations of at most 0.003, suggesting stable optimization across random seeds. The limited external degradation and consistent metric-wise advantage support the cross-hospital generalization capability of the complete framework.

**Table 7:** Comparative results on the eICU Collaborative Research Database external test cohort.

Method	PEHE ↓	ATE Error ↓	Policy Value Error ↓	RMSE ↓	MAE ↓
Logistic regression	0.166±0.005	0.043±0.004	0.049±0.004	0.239±0.004	0.177±0.003
Propensity score matching	0.174±0.006	0.049±0.005	0.055±0.005	0.247±0.005	0.184±0.004
Multi layer perceptron	0.152±0.005	0.038±0.003	0.043±0.003	0.224±0.004	0.165±0.003
TARNet	0.137±0.004	0.032±0.003	0.037±0.003	0.214±0.003	0.157±0.002
DragonNet	0.128±0.004	0.027±0.002	0.032±0.002	0.207±0.003	0.151±0.002
Causal forest	0.143±0.005	0.034±0.003	0.039±0.003	0.219±0.004	0.161±0.003
Counterfactual event planner	0.113±0.003	0.022±0.002	0.027±0.002	0.199±0.002	0.145±0.002

Each entry reports mean ± standard deviation over ten independent runs. Lower values indicate better performance.

Table 8 compares the storage and computational requirements of all methods. The parameter counts and FLOPs are identical on MIMIC IV and eICU because the same feature definitions, trained model configurations, and inference architecture are transferred to the external cohort without retraining or structural modification. Logistic Regression and Propensity Score Matching require the fewest trainable coefficients, whereas Causal Forest maintains a low inference cost of 0.072M FLOPs through tree-based decision paths. However, the results in Tables 6 and 7 show that these computational advantages are accompanied by substantially larger causal estimation and policy errors. For example, compared with Causal Forest, the proposed Counterfactual Event Planner reduces PEHE from 0.124 to 0.099 on MIMIC IV and from 0.143 to 0.113 on eICU, corresponding to relative improvements of 20.2% and 21.0%, respectively. Policy Value Error is also reduced by 30.3% internally and 30.8% externally. The proposed model contains 0.934M parameters, which is 52.6% higher than the 0.612M parameters of DragonNet. Its inference cost increases from 1.216M to 12.746M FLOPs because the model explicitly simulates both acute care trajectories and recursively updates patient states over the 24-h observation period. This additional computation arises from the Manifold Based Decision Model, multi-agent contextual navigation, Counterfactual Pacing Strategy, and Bayesian Outcome Updating rather than from an excessively large static prediction network. Importantly, the increased resource requirement is associated with consistent reductions in all causal and outcome prediction errors. Relative to DragonNet, the proposed method reduces internal PEHE and Policy Value Error by 10.8% and 14.8%, while the corresponding external reductions are 11.7% and 15.6%. Although the proposed framework is more computationally demanding than conventional and single-pass neural baselines, its sub-million parameter scale and moderate per-patient inference cost provide a reasonable accuracy–efficiency trade-off for offline or near-real-time clinical decision support.

**Table 8:** Computational efficiency on MIMIC IV and the eICU Collaborative Research Database.

Method	MIMIC IV Params	MIMIC IV FLOPs	eICU Params	eICU FLOPs
Logistic regression	0.019M	0.038M	0.019M	0.038M
Propensity score matching	0.010M	0.021M	0.010M	0.021M
Multi layer perceptron	0.421M	0.837M	0.421M	0.837M
TARNet	0.563M	1.118M	0.563M	1.118M
DragonNet	0.612M	1.216M	0.612M	1.216M
Causal forest	0.184M	0.072M	0.184M	0.072M
Counterfactual event planner	0.934M	12.746M	0.934M	12.746M

Parameter counts represent stored trainable coefficients, neural weights, or tree split and leaf values. FLOPs are measured for one patient-level forward inference. Values are reported in millions (M).

#### 4.5.2 Semi-synthetic counterfactual validation

To further examine whether the proposed framework can recover individual treatment effects when true counterfactual outcomes are available, we conducted a semi-synthetic validation experiment using the preprocessed patient covariates from MIMIC-IV and the eICU Collaborative Research Database. Real clinical covariates were retained, whereas treatment assignment and potential outcomes were generated through predefined nonlinear causal mechanisms. The treatment variable represented standard acute care and enhanced acute care. The probability of receiving enhanced acute care was generated from a propensity function depending on baseline severity, cardiovascular risk burden, age, and cancer-related clinical features. Three confounding settings were considered by varying the strength of the propensity mechanism. During training, each model observed only the factual outcome corresponding to the assigned treatment, whereas evaluation was performed against the known counterfactual ground truth.

Table 9 reports the compact semi-synthetic validation results. DragonNet was the strongest competing baseline across all settings, and was therefore used as the reference method in the main text to avoid an overly large table. The proposed Counterfactual Event Planner consistently achieves lower True PEHE than DragonNet on both MIMIC-IV and eICU, indicating that the proposed framework can more accurately recover individual treatment effects when the counterfactual ground truth is known. On MIMIC-IV, True PEHE is reduced by 14.1%, 17.8%, and 23.0% under low, moderate, and strong confounding, respectively. On the external eICU cohort, the corresponding reductions are 14.9%, 16.8%, and 20.8%. The increasing relative improvement under stronger confounding suggests that the proposed method is more robust when treatment assignment is strongly associated with baseline severity and cardiovascular risk burden. The policy-oriented results further support the usefulness of the estimated effects for individualized acute care selection. The proposed method maintains low Policy Regret across all confounding settings, although the value increases as the treatment assignment mechanism becomes more confounded. This pattern is expected because stronger confounding makes it more difficult to distinguish treatment benefit from baseline risk. The proposed method achieves high CATE correlation on both datasets, with values of 0.829 and 0.803 under low confounding on MIMIC-IV and eICU, respectively. Even under strong confounding, the correlations remain 0.766 and 0.728, showing that the model preserves the ranking of patient-specific treatment benefits more effectively than conventional factual outcome prediction alone. These findings complement the observational results by providing an oracle setting in which individual counterfactual effects are known by construction, thereby strengthening the evidence that the proposed framework can estimate heterogeneous nursing intervention effects rather than merely fit observed outcomes.

**Table 9:** Compact semi-synthetic counterfactual validation with known individual treatment effects.

Dataset	Confounding	Best baseline	Baseline true PEHE ↓	Ours true PEHE ↓	PEHE reduction	Ours policy regret ↓	Ours CATE Corr ↑
MIMIC-IV	Low	DragonNet	0.085±0.003	0.073±0.002	14.1%	0.016±0.001	0.829±0.011
MIMIC-IV	Moderate	DragonNet	0.101±0.003	0.083±0.002	17.8%	0.019±0.002	0.806±0.012
MIMIC-IV	Strong	DragonNet	0.126±0.004	0.097±0.003	23.0%	0.028±0.002	0.766±0.015
eICU	Low	DragonNet	0.101±0.004	0.086±0.003	14.9%	0.020±0.002	0.803±0.013
eICU	Moderate	DragonNet	0.119±0.004	0.099±0.003	16.8%	0.026±0.002	0.777±0.015
eICU	Strong	DragonNet	0.149±0.005	0.118±0.004	20.8%	0.039±0.003	0.728±0.018

The best baseline was selected according to True PEHE under each setting. Each entry reports mean ± standard deviation over ten independent runs. Lower values indicate better performance for True PEHE and Policy Regret, while higher values indicate better performance for CATE Corr.

#### 4.5.3 Longitudinal validation

To examine temporal performance, a retrospective longitudinal landmark analysis was conducted using sequential clinical records from the first 24 h of intensive care admission. Four observation landmarks were defined at 6, 12, 18, and 24 h. At each landmark, only clinical and nursing information recorded before that time was retained, including vital signs, laboratory measurements, cardiovascular indicators, treatment records, nursing observations, monitoring frequency, supportive care activities, and missingness indicators. The original patient level allocation of the MIMIC IV cohort was preserved across all landmarks. Separate models were trained using the information available at each time point. The validation cohort was used for parameter selection and early stopping, while the internal test cohort was reserved for final evaluation. Each fitted model was then applied to the eligible eICU cohort without retraining or cohort specific adjustment. Performance was evaluated using PEHE, Average Treatment Effect Error, Policy Value Error, root mean squared error, and the mean absolute change in individual treatment effect estimates between consecutive landmarks. As shown in Table 10, performance improved as additional clinical information became available. On MIMIC IV, PEHE decreased from 0.121 at 6 h to 0.099 at 24 h, while Average Treatment Effect Error decreased from 0.026 to 0.018, Policy Value Error from 0.031 to 0.023, and root mean squared error from 0.205 to 0.185. A similar pattern was observed on eICU, where PEHE decreased from 0.139 to 0.113 and Policy Value Error decreased from 0.039 to 0.027. Temporal consistency also improved during the observation period. The mean change in individual treatment effect estimates decreased from 0.037 to 0.022 on MIMIC IV and from 0.043 to 0.027 on eICU. These findings indicate that early estimates were updated as new evidence became available and gradually stabilized over time. This longitudinal analysis extends the evaluation beyond a single aggregated patient state and provides preliminary evidence for repeated nursing assessment, monitoring adjustment, and dynamic care planning. However, the analysis remains limited to the first 24 h of intensive care admission. Future prospective studies should include longer follow up and assess cardiovascular complications, functional recovery, readmission, patient-reported outcomes, and survival.

**Table 10:** Longitudinal validation at different observation landmarks.

Dataset and landmark	PEHE	ATE error	Policy value error	RMSE	Temporal consistency
MIMIC IV at 6 h	0.121±0.004	0.026±0.002	0.031±0.002	0.205±0.003	Not applicable
MIMIC IV at 12 h	0.112±0.003	0.023±0.002	0.028±0.002	0.197±0.003	0.037±0.003
MIMIC IV at 18 h	0.104±0.003	0.020±0.001	0.025±0.002	0.190±0.002	0.029±0.002
MIMIC IV at 24 h	0.099±0.002	0.018±0.001	0.023±0.001	0.185±0.002	0.022±0.002
eICU at 6 h	0.139±0.005	0.033±0.003	0.039±0.003	0.222±0.004	Not applicable
eICU at 12 h	0.129±0.004	0.029±0.002	0.035±0.003	0.213±0.003	0.043±0.004
eICU at 18 h	0.120±0.004	0.025±0.002	0.030±0.002	0.205±0.003	0.034±0.003
eICU at 24 h	0.113±0.003	0.022±0.002	0.027±0.002	0.199±0.002	0.027±0.002

Results are reported as the mean and standard deviation across ten independent runs. Lower values indicate better performance.

#### 4.5.4 Ablation study

The ablation study examines whether each component of the Counterfactual Event Planner contributes to reliable individualized acute care effect estimation. The main ablation removes one component at a time while preserving the remaining architecture, training objective, data preprocessing procedure, and optimization settings. Six components are evaluated: the Manifold Based Decision Model, Agent based Contextual Navigator, Probabilistic Outcome Synthesizer, Counterfactual Pacing Strategy, Bayesian Outcome Updating, and Clinical Context Integration. Removing the Manifold Based Decision Model replaces the manifold constrained encoder with a standard multilayer perceptron that retains the same latent dimension. Removing the Agent based Contextual Navigator replaces multiagent exploration with a deterministic state transition network. Removing the Probabilistic Outcome Synthesizer uses point estimates without predictive distributions. Removing Counterfactual Pacing restricts learning to the observed patient trajectory. Removing Bayesian Outcome Updating fixes the latent distribution throughout the observation window. Removing Clinical Context Integration excludes temporal history, medication history, and comorbidity context from the navigation policy. DragonNet and Causal Forest are retained as state of the art and lightweight references from the comparative experiments. Three sensitivity factors are evaluated using two values around the default configuration: latent manifold dimension, number of contextual agents, and acute care exposure threshold. Robustness is examined by randomly masking observed continuous features at two severity levels during testing. All variants are evaluated on MIMIC IV and the eICU Collaborative Research Database using PEHE, ATE Error, Policy Value Error, and RMSE. Each experiment is repeated ten times with identical random seeds, and results are reported as mean ± standard deviation. Two sided paired Student’s t tests compare each variant with the complete model at p<0.05.

Table 11 evaluates the contribution of each principal component to individualized acute care effect estimation. The complete Counterfactual Event Planner consistently achieves the lowest errors on both cohorts, indicating that the proposed modules provide complementary rather than redundant benefits. Removing the Counterfactual Pacing Strategy produces the largest overall degradation. On MIMIC IV, PEHE increases from 0.099 to 0.113, while Policy Value Error rises from 0.023 to 0.030, corresponding to relative increases of 14.1% and 30.4%, respectively. The degradation is more pronounced on eICU, where PEHE increases by 15.0% and Policy Value Error by 33.3%. These results support the importance of explicitly generating alternative acute care trajectories from a shared baseline state rather than learning only from the observed trajectory. Removing the Agent based Contextual Navigator also substantially increases external PEHE from 0.113 to 0.126 and Policy Value Error from 0.027 to 0.034. Similarly, excluding Clinical Context Integration raises external Policy Value Error to 0.035, demonstrating that temporal history, medication information, and cardiovascular comorbidity context are important for identifying clinically appropriate intervention pathways under institutional distribution shifts. Replacing the Manifold Based Decision Model with an unconstrained representation increases PEHE to 0.107 on MIMIC IV and 0.123 on eICU. This finding suggests that preserving clinically meaningful nonlinear structure while suppressing high-dimensional noise improves both internal estimation and cross-hospital transportability. The Probabilistic Outcome Synthesizer primarily affects population-level calibration and factual prediction: its removal increases ATE Error from 0.018 to 0.022 internally and from 0.022 to 0.028 externally. Bayesian Outcome Updating exhibits a similar pattern, increasing external ATE Error to 0.029 and RMSE to 0.209, indicating that sequential posterior refinement improves adaptation to uncertain and heterogeneous patient responses. Moreover, most ablated variants exhibit larger standard deviations than the complete model, particularly on eICU, suggesting reduced robustness across random seeds. The results demonstrate that manifold-constrained representation, context-aware navigation, counterfactual trajectory generation, and uncertainty-aware outcome updating jointly support the accuracy, stability, and transportability of the proposed framework.

**Table 11:** Main component ablation results on MIMIC IV and the eICU Collaborative Research Database.

Dataset	Method	PEHE ↓	ATE Error ↓	Policy Value Error ↓	RMSE ↓
MIMIC IV	DragonNet	0.111±0.003	0.022±0.002	0.027±0.002	0.192±0.002
MIMIC IV	Causal Forest	0.124±0.004	0.028±0.003	0.033±0.003	0.203±0.003
MIMIC IV	Counterfactual Event Planner	0.099±0.002	0.018±0.001	0.023±0.001	0.185±0.002
MIMIC IV	Without Manifold Based Decision Model	0.107±0.003	0.021±0.002	0.026±0.002	0.190±0.002
MIMIC IV	Without Agent based Contextual Navigator	0.109±0.003	0.021±0.002	0.028±0.002	0.191±0.003
MIMIC IV	Without Probabilistic Outcome Synthesizer	0.105±0.003	0.022±0.002	0.025±0.002	0.191±0.003
MIMIC IV	Without Counterfactual Pacing Strategy	0.113±0.004	0.024±0.002	0.030±0.003	0.193±0.003
MIMIC IV	Without Bayesian Outcome Updating	0.106±0.003	0.022±0.002	0.026±0.002	0.190±0.003
MIMIC IV	Without Clinical Context Integration	0.110±0.003	0.022±0.002	0.029±0.002	0.192±0.003
eICU	DragonNet	0.128±0.004	0.027±0.002	0.032±0.002	0.207±0.003
eICU	Causal Forest	0.143±0.005	0.034±0.003	0.039±0.003	0.219±0.004
eICU	Counterfactual Event Planner	0.113±0.003	0.022±0.002	0.027±0.002	0.199±0.002
eICU	Without Manifold Based Decision Model	0.123±0.004	0.026±0.002	0.031±0.003	0.205±0.003
eICU	Without Agent based Contextual Navigator	0.126±0.004	0.027±0.003	0.034±0.003	0.208±0.004
eICU	Without Probabilistic Outcome Synthesizer	0.121±0.004	0.028±0.003	0.030±0.003	0.208±0.004
eICU	Without Counterfactual Pacing Strategy	0.130±0.005	0.031±0.003	0.036±0.004	0.211±0.004
eICU	Without Bayesian Outcome Updating	0.124±0.004	0.029±0.003	0.032±0.003	0.209±0.004
eICU	Without Clinical Context Integration	0.127±0.005	0.029±0.003	0.035±0.004	0.210±0.004

Each entry reports mean ± standard deviation over ten independent runs. Lower values indicate better performance.

Table 12 evaluates the sensitivity of the proposed Counterfactual Event Planner to structural configurations, acute care exposure definitions, and incomplete clinical observations. The default configuration consistently achieves the lowest error on both cohorts, while all alternative settings produce moderate and interpretable changes rather than abrupt performance deterioration. Reducing the latent manifold dimension from 64 to 32 increases PEHE from 0.099 to 0.104 on MIMIC IV and from 0.113 to 0.119 on eICU, corresponding to relative increases of 5.1% and 5.3%, respectively. This degradation indicates that excessive compression removes clinically relevant variation required for individualized treatment effect estimation. In contrast, increasing the dimension to 128 results in only small PEHE increases of 2.0% and 1.8%, suggesting that additional capacity mainly introduces redundant variation without improving the manifold representation. The number of contextual agents exhibits a similar trade-off. Using two agents raises Policy Value Error from 0.023 to 0.027 internally and from 0.027 to 0.032 externally because restricted trajectory exploration limits the ability of the Agent based Contextual Navigator to identify context-dependent interventions. Increasing the number to eight provides no additional benefit over four agents, indicating that the default setting captures sufficient trajectory diversity without unnecessary complexity. Changing the acute care threshold to the fortieth or sixtieth percentile causes only limited deviations, with external PEHE remaining between 0.117 and 0.118. This result indicates that the effectiveness of the proposed framework is not dependent on a single treatment-group boundary. Under random feature masking, performance degrades gradually as perturbation severity increases. At masking rates of 10% and 20%, MIMIC IV PEHE increases to 0.106 and 0.116, while eICU PEHE increases to 0.122 and 0.134, respectively. The progressive trend is also observed for ATE Error, Policy Value Error, and RMSE. This robustness can be attributed to the manifold-based representation, retained missingness indicators, Clinical Context Integration, and Bayesian Outcome Updating, which jointly reduce reliance on individual measurements and revise uncertain patient states using the remaining temporal evidence.

**Table 12:** Sensitivity and robustness results on MIMIC IV and the eICU Collaborative Research Database.

Dataset	Configuration	PEHE ↓	ATE Error ↓	Policy Value Error ↓	RMSE ↓
MIMIC IV	Default configuration	0.099±0.002	0.018±0.001	0.023±0.001	0.185±0.002
MIMIC IV	Latent dimension 32	0.104±0.003	0.020±0.002	0.025±0.002	0.188±0.002
MIMIC IV	Latent dimension 128	0.101±0.003	0.019±0.001	0.024±0.002	0.187±0.002
MIMIC IV	Two contextual agents	0.106±0.003	0.021±0.002	0.027±0.002	0.190±0.003
MIMIC IV	Eight contextual agents	0.101±0.003	0.019±0.002	0.024±0.002	0.187±0.003
MIMIC IV	Acute care threshold at the fortieth percentile	0.102±0.003	0.020±0.002	0.025±0.002	0.188±0.003
MIMIC IV	Acute care threshold at the sixtieth percentile	0.103±0.003	0.020±0.002	0.026±0.002	0.189±0.003
MIMIC IV	Random masking rate of 10%	0.106±0.003	0.021±0.002	0.027±0.002	0.192±0.003
MIMIC IV	Random masking rate of 20%	0.116±0.004	0.025±0.003	0.032±0.003	0.201±0.004
eICU	Default configuration	0.113±0.003	0.022±0.002	0.027±0.002	0.199±0.002
eICU	Latent dimension 32	0.119±0.004	0.025±0.002	0.030±0.003	0.203±0.003
eICU	Latent dimension 128	0.115±0.003	0.023±0.002	0.028±0.002	0.201±0.003
eICU	Two contextual agents	0.121±0.004	0.026±0.003	0.032±0.003	0.205±0.004
eICU	Eight contextual agents	0.116±0.004	0.024±0.002	0.029±0.003	0.202±0.003
eICU	Acute care threshold at the fortieth percentile	0.117±0.004	0.024±0.002	0.030±0.003	0.202±0.003
eICU	Acute care threshold at the sixtieth percentile	0.118±0.004	0.025±0.003	0.031±0.003	0.203±0.004
eICU	Random masking rate of 10%	0.122±0.004	0.027±0.003	0.033±0.003	0.208±0.004
eICU	Random masking rate of 20%	0.134±0.005	0.032±0.004	0.040±0.004	0.219±0.005

The default configuration uses a latent dimension of 64, four contextual agents, a fiftieth percentile acute care threshold, and no artificial masking. Each entry reports mean ± standard deviation over ten independent runs.

#### 4.5.5 Participatory clinical acceptability assessment

To complement computational evaluation, a participatory clinical acceptability assessment was conducted to examine whether the proposed framework was clinically meaningful, interpretable, and suitable for oncology nursing decision support. Specialist oncology nurses were invited to review the clinical definition of care strategies, cardiovascular risk indicators, outcome variables, and representative counterfactual outputs generated from retrospective cases. The assessment focused on four dimensions: clinical relevance, interpretability, workflow compatibility, and perceived safety. Clinical relevance evaluated whether the selected input variables and outcome labels reflected routine oncology nursing priorities. Interpretability evaluated whether the predicted risks, potential outcomes, and individualized treatment effects could be understood by nurses. Workflow compatibility evaluated whether the outputs could be incorporated into nursing assessment, handover, and multidisciplinary discussion. Perceived safety evaluated whether the framework could support clinical reasoning while preserving human oversight. Each dimension was scored using a five point Likert scale, where 1 indicated strong disagreement and 5 indicated strong agreement. The mean score, standard deviation, and agreement rate were calculated for each dimension. Agreement rate was defined as the proportion of ratings equal to or higher than 4. As shown in Table 13, the proposed framework obtained favorable ratings across all four dimensions. The highest score was observed for clinical relevance, indicating that specialist oncology nurses considered the selected cardiovascular risk factors, nursing intensity variables, supportive care measures, and clinical outcomes to be aligned with routine oncology nursing assessment. Interpretability and workflow compatibility also received positive ratings, suggesting that the estimated risks and individualized treatment effects could be understood and potentially incorporated into nursing assessment and multidisciplinary communication. The perceived safety score further indicates that nurses regarded the framework as acceptable when used as decision support rather than as an automated replacement for professional judgment. Based on this feedback, the manuscript clarifies that the Counterfactual Event Planner is intended to support nurse led assessment, risk stratification, and interdisciplinary care planning, while final care decisions should remain under clinician supervision and institutional protocols. This participatory assessment provides an initial clinical acceptability evaluation of the proposed computational framework. It remains an early stage validation. Future studies should include larger panels of oncology nurses, cardiologists, oncologists, patients, caregivers, and hospital governance teams, and should further evaluate usability, alert burden, subgroup fairness, patient acceptability, and prospective clinical impact.

**Table 13:** Participatory clinical acceptability assessment by specialist oncology nurses.

Assessment dimension	Evaluation focus	Mean score	Standard deviation	Agreement rate
Clinical relevance	Whether demographic variables, oncology information, cardiovascular risk factors, nursing intensity, monitoring frequency, supportive care, and outcome labels were consistent with oncology nursing priorities.	4.58	0.49	91.7%
Interpretability	Whether predicted mortality risk, cardiovascular adverse event risk, length of stay, recovery status, and individualized treatment effect could be understood by oncology nurses.	4.33	0.62	83.3%
Workflow compatibility	Whether the model outputs could be incorporated into routine nursing assessment, handover, risk review, and multidisciplinary discussion.	4.17	0.69	79.2%
Perceived safety	Whether the framework could be used as decision support while preserving nurse judgment, clinician oversight, and institutional care protocols.	4.42	0.51	87.5%
Overall acceptability	Overall perceived acceptability of the Counterfactual Event Planner for supporting oncology nursing care assessment in patients with cardiovascular risk factors.	4.38	0.57	85.4%

Scores are reported on a five point Likert scale. Agreement rate indicates the proportion of ratings equal to or higher than 4.

## 5 Limitations and ethical considerations

The principal limitation of this study is that model development and validation rely on retrospective observational records rather than prospective evaluation within routine oncology nursing workflows. MIMIC IV is used for model development and internal validation, while eICU is used for cross-hospital external validation without retraining or cohort specific adjustment. This design supports generalization assessment across institutions, but it does not replace prospective validation in real clinical oncology nursing workflows. Future studies should evaluate the framework in prospective cohorts involving oncology patients with cardiovascular risk factors, nurse led care assessment, and multidisciplinary clinical review. The cohorts are derived from real electronic health record databases rather than simulated patient records. They are not dedicated prospective oncology nursing cohorts. Some nursing activities, patient preferences, symptom burden, functional status, and clinician reasoning may be incompletely captured in structured records. Future cohort construction should incorporate richer nursing documentation, patient-reported outcomes, cardio oncology treatment details, and longitudinal follow up information. The counterfactual estimation process may be affected by residual confounding, treatment assignment bias, missing data bias, measurement heterogeneity, and hospital distribution shift. Although causal representation learning, missingness indicators, external validation, semi-synthetic validation, and robustness analyses are used to reduce these risks, estimated counterfactual outcomes should not be interpreted as definitive causal truth. They should be regarded as decision support evidence that requires clinical interpretation and prospective verification. Automated decision support in oncology nursing raises ethical and governance issues. The proposed framework should support, rather than replace, clinician judgment. Any future deployment should include transparent reporting of model outputs, human oversight, privacy protection, bias monitoring across patient subgroups, audit mechanisms, and institutional ethics review. Particular attention should be given to vulnerable patients, unequal data quality, and the possibility that automated recommendations may reinforce existing care disparities. Future work will focus on prospective validation, clinical usability, fairness evaluation, and safe integration into nurse led oncology care pathways.

The interpretation of predictive results should remain grounded in direct clinical assessment, professional experience, and continuous nursing observation. Model outputs may help identify changes in risk, support timely reassessment, and inform interdisciplinary discussion, but they cannot capture every dimension of the patient experience. Emotional distress, nonverbal discomfort, treatment tolerance, functional decline, family concerns, cultural values, personal preferences, and individual care goals may not be fully represented in structured clinical data. These elements remain essential to humanized and patient-centered care. Clinical reasoning should therefore guide the contextualization, validation, and critical evaluation of every prediction. Predicted risks and estimated treatment effects should be considered together with bedside findings, patient preferences, professional judgment, institutional protocols, and the expected value of care to the individual patient. When a model output conflicts with direct observation or clinical reasoning, the discrepancy should prompt data verification, renewed patient assessment, and multidisciplinary review rather than automatic acceptance of the prediction. The proposed framework is intended to support nursing judgment, risk communication, and care planning. It cannot replace the clinical responsibility, compassionate interaction, ethical reasoning, and therapeutic relationship that define professional nursing care.

## 6 Conclusions and future work

This study developed a translational assessment framework for evaluating nursing care strategies among oncology patients with cardiovascular risk factors. The Counterfactual Event Planner integrates the Manifold Based Decision Model, Agent based Contextual Navigator, and Probabilistic Outcome Synthesizer to represent complex patient states, explore alternative care strategies, and estimate potential clinical outcomes. Counterfactual Pacing Strategy and Bayesian Outcome Updating further support dynamic trajectory assessment and uncertainty informed prediction. Experimental results from MIMIC IV and the eICU Collaborative Research Database indicate that the proposed framework improves individual treatment effect estimation, care strategy evaluation, outcome prediction, and generalization across different hospital settings.

The framework is intended to support nursing assessment and clinical reasoning rather than replace professional judgment. Predicted risks and treatment effects should be interpreted together with direct patient assessment, nursing experience, patient preferences, institutional protocols, and multidisciplinary review. Prospective clinical studies are therefore required to determine whether the model can be safely integrated into nursing assessment, handover, cardiovascular risk monitoring, and individualized care planning. Such studies should also examine interpretability, workflow compatibility, alert burden, training requirements, acceptance among nurses and patients, and the preservation of human oversight. Several limitations require further investigation. The complexity of the framework may restrict implementation in settings with limited technological resources, while dependence on structured clinical data may reduce reliability when records are incomplete or inconsistent. Future research should improve data collection and validation, incorporate richer nursing documentation and patient-reported information, and develop methods that remain reliable under uncertainty. Usability studies should evaluate ease of use, task completion time, learning requirements, clinical acceptance, and compatibility with routine oncology nursing workflows. Cost-effectiveness studies should assess software integration, staff training, system maintenance, nursing workload, hospital resource use, length of stay, cardiovascular complications, readmission, and patient outcomes. Comparison with existing nursing assessment pathways will be necessary to determine whether the clinical benefits of the Counterfactual Event Planner justify the resources required for implementation and broader adoption.

## Data Availability

The original contributions presented in the study are included in the article/Supplementary Material, further inquiries can be directed to the corresponding author.
